# The Genetic Dissection of Nitrogen Use-Related Traits in Flax (*Linum usitatissimum* L.) at the Seedling Stage through the Integration of Multi-Locus GWAS, RNA-seq and Genomic Selection

**DOI:** 10.3390/ijms242417624

**Published:** 2023-12-18

**Authors:** Braulio J. Soto-Cerda, Giovanni Larama, Sylvie Cloutier, Bourlaye Fofana, Claudio Inostroza-Blancheteau, Gabriela Aravena

**Affiliations:** 1 Departamento de Ciencias Agropecuarias y Acuícolas, Universidad Católica de Temuco, Rudecindo Ortega 02950, Temuco 4781312, Chile; claudio.inostroza@uct.cl (C.I.-B.); gaby.aravena@gmail.com (G.A.); 2 Núcleo de Investigación en Producción Alimentaria, Facultad de Recursos Naturales, Universidad Católica de Temuco, Rudecindo Ortega 02950, Temuco 4781312, Chile; 3 Center of Plant, Soil Interaction and Natural Resources Biotechnology, Scientific and Technological Bioresource Nucleus, Universidad de La Frontera, Temuco 4811230, Chile; giovanni.larama@ufrontera.cl; 4 Biocontrol Research Laboratory, Universidad de La Frontera, Temuco 4811230, Chile; 5 Ottawa Research and Development Centre, Agriculture and Agri-Food Canada, 960 Carling Avenue, Ottawa, ON K1A 0C6, Canada; sylviej.cloutier@agr.gc.ca; 6 Charlottetown Research and Development Centre, Agriculture and Agri-Food Canada, 440 University Avenue, Charlottetown, PE C1A 4N6, Canada

**Keywords:** *Linum usitatissimum*, nitrogen use efficiency, GWAS, RNA-seq, genomic selection

## Abstract

Nitrogen (N), the most important macro-nutrient for plant growth and development, is a key factor that determines crop yield. Yet its excessive applications pollute the environment and are expensive. Hence, studying nitrogen use efficiency (NUE) in crops is fundamental for sustainable agriculture. Here, an association panel consisting of 123 flax accessions was evaluated for 21 NUE-related traits at the seedling stage under optimum N (N+) and N deficiency (N−) treatments to dissect the genetic architecture of NUE-related traits using a multi-omics approach integrating genome-wide association studies (GWAS), transcriptome analysis and genomic selection (GS). Root traits exhibited significant and positive correlations with NUE under N− conditions (r = 0.33 to 0.43, *p* < 0.05). A total of 359 QTLs were identified, accounting for 0.11% to 23.1% of the phenotypic variation in NUE-related traits. Transcriptomic analysis identified 1034 differentially expressed genes (DEGs) under contrasting N conditions. DEGs involved in N metabolism, root development, amino acid transport and catabolism and others, were found near the QTLs. GS models to predict NUE stress tolerance index (NUE_STI) trait were tested using a random genome-wide SNP dataset and a GWAS-derived QTLs dataset. The latter produced superior prediction accuracy (r = 0.62 to 0.79) compared to the genome-wide SNP marker dataset (r = 0.11) for NUE_STI. Our results provide insights into the QTL architecture of NUE-related traits, identify candidate genes for further studies, and propose genomic breeding tools to achieve superior NUE in flax under low N input.

## 1. Introduction

Nitrogen (N) is the most important mineral nutrient for plant growth and development, and it is key in determining yield in non-N-fixing crops [1]. In the last four decades, a twofold increase in food production was only achieved through a disproportionate sevenfold increase in the use of N fertilizer [2]. In 2018, 186 million tons of N fertilizer were utilized globally [3]. Given the world’s projected human population by 2050, a further threefold increase in N input will be needed to fulfill the global food demand [4]. Although N has the most direct impact on crop production, plants can only uptake and use 30–40% of the applied N [5]. The unused N, lost in the atmosphere and through groundwater and rivers, causes environmental pollution [5,6]. Moreover, chemical N production greatly depends on fossil energy, contributing to additional greenhouse emissions and raising fertilizer costs [7]. Generally, low N use efficiency (NUE) in crops leads to excessive use of N fertilizer, which is not environment-friendly or cost-effective for food production [3,5].

NUE is defined as crop yield per unit of available N in the soil [8] and consists of two main components: N uptake efficiency (NupE), which includes traits such as root architecture and transporter activity; and N utilization efficiency (NutE), which includes all the processes related to the plant’s capacity to assimilate and remobilize N into the seeds [9,10]. It is of strategic importance to improve crops’ NUE because a mere 1% increase in NUE would translate into ~$2.3 B USD annual savings in N fertilizer costs, while also reducing the environmental footprint [11].

Plants uptake N from the soil mainly as nitrate (NO_3_^−^), ammonium (NH_4_^+^) or as amino acids under specific soil conditions [12,13]. A plant’s response to N involves multiple genes that control root/shoot growth, N metabolism, and N content. In *Arabidopsis*, four families of nitrate transporters, *NRT1*, *NRT2*, *CLC* and *SLAC/SLAH*, have been characterized by Masclaux-Daubresse et al. [9]. Among the *NRT1* gene family, *NRT1.1* plays roles in NO_3_^−^ uptake and translocation from roots to shoots [14]. In addition to *NRT1.1*, *NRT1.2* and *NAXT1* have also been reported to be involved in NO_3_^−^ uptake with *NRT1.2* and *NAXT1* ensuring influx and efflux activities, respectively [15]. *NRT1.8*, *NRT1.5* and *NRT1.9* were described as major transporters for long-distance NO_3_^−^ distribution. *NRT1.8* is known to modulate NO_3_^−^ loading into the xylem parenchyma cells within the vascular system [16], whereas *NRT1.9* mediates NO_3_^−^ loading into the root phloem, thereby influencing NO_3_^−^ translocation and distribution [17]. *NRT1.11* and *NRT1.12* are expressed in mature leaves and mediate NO_3_^−^ distribution to young tissues [18]. Once NO_3_^−^ is absorbed by roots, it is first reduced to nitrite (NO_2_^−^) by a cytosolic nitrate reductase (NR), and then to NH_4_^+^ by a nitrite reductase (NiR) [19]. Together with glutamate synthase (GOGAT), glutamine synthetase (GS), a key enzyme in N assimilation and remobilization, forms the GS-GOGAT cycle in which inorganic NH_4_^+^ is converted into glutamine [19]. In addition to the transport and assimilation genes, many transcription factors are involved in the regulation of NUE (reviewed by Masclaux-Daubresse [9]; Hudson [20]; Alvarez [21]; Luo [13]). The extensive nature of this gene network illustrates the complexity of N response, NUE regulation, and its underlying genetic mechanisms, making conventional plant breeding progress slow and success not guaranteed.

A variety of traits contributing to NUE in plants have been evaluated in many bi-parental populations and the associated QTL studies have been reported [22,23,24]. At the seedling stage, QTL studies have focused on NUE-related traits and their associations with root system architecture (RSA) traits. In maize, 184 NUE-related QTLs and 147 RSA-related QTLs were identified [10]. Similarly, five and six stable pleiotropic QTL influencing RSA-NUE traits were reported in rapeseed and wheat, respectively. In the adult plant stage, QTL studies have focused on yield-related traits, such as grain and biomass N content, physiological traits and their correlations to NupE and NutE [22,24,25]. In maize, Hirel [25] identified one QTL that collocated with NUE, yield, nitrate content, nitrate reductase and glutamine synthetase activities, and suggested a co-regulation of these traits.

Recently, genome-wide association studies (GWAS) have become a benchmark approach for fine-mapping QTL and postulating candidate genes. GWAS models are powerful tools for detecting QTLs by taking advantage of historical recombination events accumulated in diverse germplasm collections [26]. For NUE-related traits, GWAS have been conducted in wheat (*Triticum aestivum*) [27,28], winter rapeseed (*Brassica napus*) [7], maize [29] and Indian mustard [30]. In rapeseed seedlings, 14 QTL clusters were associated with 13 root- and biomass-related traits and NUE [31].

Whereas GWAS can identify putative genes associated with its detected QTLs, the gene expression patterns require further validation through gene expression studies, and RNA sequencing (RNAseq) is a prime approach for such a validation. By combining GWAS and RNA-seq data, gene expression patterns for putative functional genes located within GWAS-identified QTL intervals have been reported and the involved biological processes more strongly validated than either strategy taken alone [32,33,34]. By cross-referencing the DEGs and GWAS-derived QTL-associated candidate genes, functional genes associated with harvest index-related traits in *Brassica napus* (rapeseed) [34], genes that co-modulate root traits and yield under drought in flax (*Linum usitatissimum*) [35], and genes that regulate root growth and NUE under depleted N conditions in rapeseed [31] have been reported.

Genomic selection (GS) is a strategy that is well suited to improve quantitative traits, particularly those with low heritability, as it makes use of all marker effects across the genome to calculate genomic estimated breeding values (GEBVs) for individuals’ selection [36]. In traditional GS, prediction models of phenotypic traits using genome-wide markers are constructed based on a training population and then applied to test populations [37]. Using these models, the genetic effect values of unobserved individuals are predicted, allowing the use of some small-effect markers that would otherwise remain undetected in GWAS. An alternative strategy to genome-wide markers to predict GEBVs is to make use of markers obtained from GWAS, a variation of GS known as GWAS-assisted GS. GWAS removes a large proportion of unrelated markers, resulting in a limited number of favorable genetic loci linked to the trait of interest [38]. The predictive ability of GWAS-derived markers has been reported to be similar or superior to that attained using genome-wide markers in rice, citrus and flax [39,40,41,42].

Flax (*Linum usitatissimum* L.) is an important cash crop traditionally used as a natural fiber or oilseed crop with many health promoting attributes [43]. Its seed contains 40–45% oil, 20–25% protein, 20% insoluble fiber, 10% soluble fiber (mucilage) and 1% lignan secoisolariciresinol diglucosides (SDGs) [44]. Aside from being a rich source of functional ingredients, flax is a profitable option for many crop rotation scenarios. High-yielding flax production is achieved with 100–150 kg ha-1 of N, accounting for 35–50% of its total production costs [45]. Therefore, an increased flax NUE will likely enhance its competitiveness with other crops, while minimizing its environmental footprint. Genetic variation in N accumulation, translocation and NUE has been reported in flax, and a moderate N supply (50–90 kg ha^−1^) was found to be adequate for promoting a maximum N translocation, NUE and oil production [46,47]. Whereas the detection of NUE variation in flax is promising, studies using genomic approaches including GWAS, RNA-seq and GS to decipher the genetic architecture of NUE have so far not been reported.

The objectives of the current study were to: (i) identify genetic loci associated with 21 NUE-related traits under optimal and deficient N conditions during the seedling stage of a flax association panel; (ii) generate and combine GWAS and RNA-seq data to identify candidate genes associated with root response under starved N conditions; and (iii) compare QTL markers and genome-wide SNPs for their GS prediction accuracies for NUE_STI dueing the seedling stage in flax.

## 2. Results

### 2.1. Variations in Root and Biomass Phenotypic Traits

**Figure 1:**
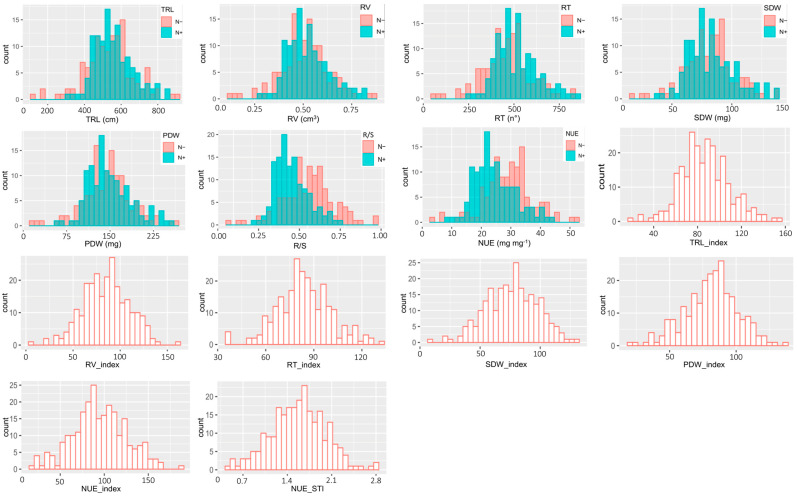
Phenotypic distribution of 21 NUE-related traits in flax including root and biomass traits, and trait indices under depleted N (N−) and optimum N (N+) conditions.

The REML analysis showed that N treatment and genotype were significant (*p* < 0.001) for all traits except for the root dry weight (Table S2). The interaction between treatment and genotype was only found to be significant for shoot dry weight. Plants grown under N+ condition had statistically higher mean values for all traits, except for root/shoot ratio (R/S) and NUE, which showed higher mean values under N− conditions (Table 1). The R/S was 0.490 under N− treatment whereas plants grown under N+ had 0.407 as an R/S. The trait stability indices ranged from 76.1% (SDW_Index) to 87.3% (TRL_Index). The coefficients of variation for all traits were higher in the N− than under N+ treatment and ranged from 27.8 to 64.0% (Table 1). In general, the 21 NUE-related traits were normally distributed or close to normality (Figure 1).

Moderate to high (*p* < 0.05) correlation coefficients (r) were observed between the root and biomass traits, and the stability indices under both N treatments ranged from 0.18 to 0.99 (Figure S1). Some stability indices were significantly (*p* < 0.05) and negatively correlated with R/S_N+, R/S_N− and NUE_N+. Root traits showed significant and positive correlations with NUE and NUE_STI under both N conditions (r = 0.18 to 0.52, *p* < 0.05). Root volume (RV_N−) and R/S_N− were the most contributing traits to higher NUE under N stress, while R/S_N− and RV_N+ accounted for the most variation observed for NUE_N+ (Figure S1). In contrast, biomass traits (SDW and PDW) did not show any significant correlation with NUE_N−, NUE_N+ and NUE_STI.

### 2.2. Genetic Structure and Linkage Disequilibrium

**Figure 2:**
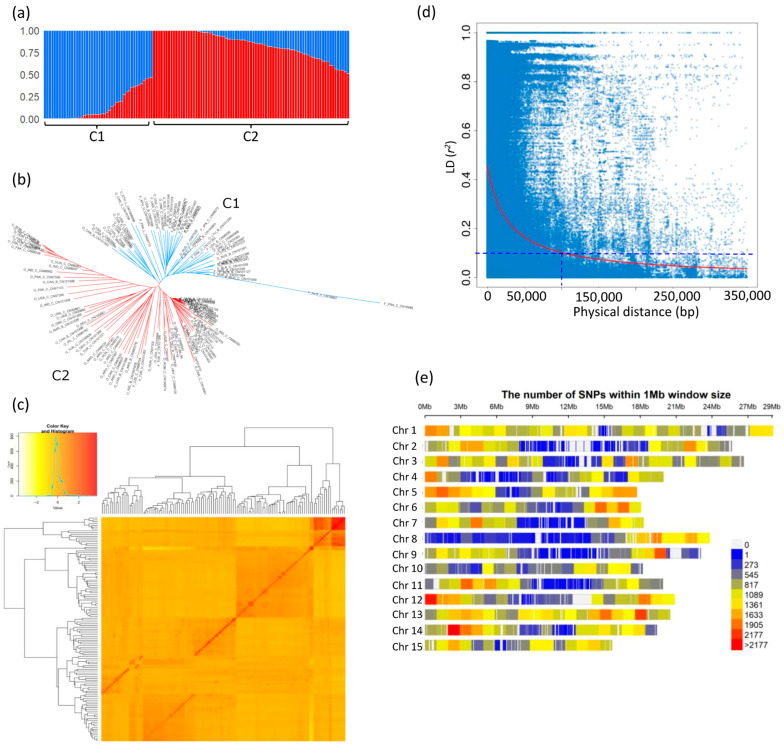
(**a**) Model-based population structure of 123 flax accessions belonging to two clusters predefined by the STRUCTURE software. (**b**) Neighbor-joining (NJ) tree of 123 flax accessions based on the 272,944 SNPs. (**c**) Kinship relationships among the 123 flax accessions of the collection. (**d**) Genome-wide linkage disequilibrium (LD) decay of *r*^2^ values (red line), against physical distance (bp) in flax. The dashed blue line indicates the cutoff value (*r*^2^ = 0.1) used to determine the genome-wide LD block size. (**e**) Distribution of 272,944 SNPs used for GWAS analysis across the 15 *Linum usitatissimum* chromosomes. C1: cluster 1; C2: cluster 2.

The genetic structure analysis grouped the 123 flax accessions into two clusters (C1 and C2) as revealed by STRUCTURE (Figure 2a). The neighbor-joining (NJ) clustering and STRUCTURE produced similar outcomes, with the C2 accessions being the largest cluster (Figure 2b).

The kinship heatmap based on the identity by state (IBS) values showed the presence of three major clusters (Figure 2c). In general, intra group kinship was stronger than inter group kinship relationships among the 123 accessions, and the inter group kinship values were weaker than the intra group relationships.

The genome-wide LD decay was estimated to be 100 kb (*r*^2^ = 0.1) (Figure 2d). The chromosome-specific LD decays (*r*^2^ = 0.1) varied from 50 kb on chromosomes 4, 7 and 8 to 200 kb on chromosome 13 (Figure S2). The LD decay used for grouping QTNs into QTL (*r*^2^ > 0.3) for chromosomes harboring QTNs ranged from 10 kb on chromosome 4 to 40 kb on chromosome 13 with a mean of 14.3 kb (Figure S2).

### 2.3. ML-GWAS of 21 NUE-Related Traits in Flax

**Figure 3:**
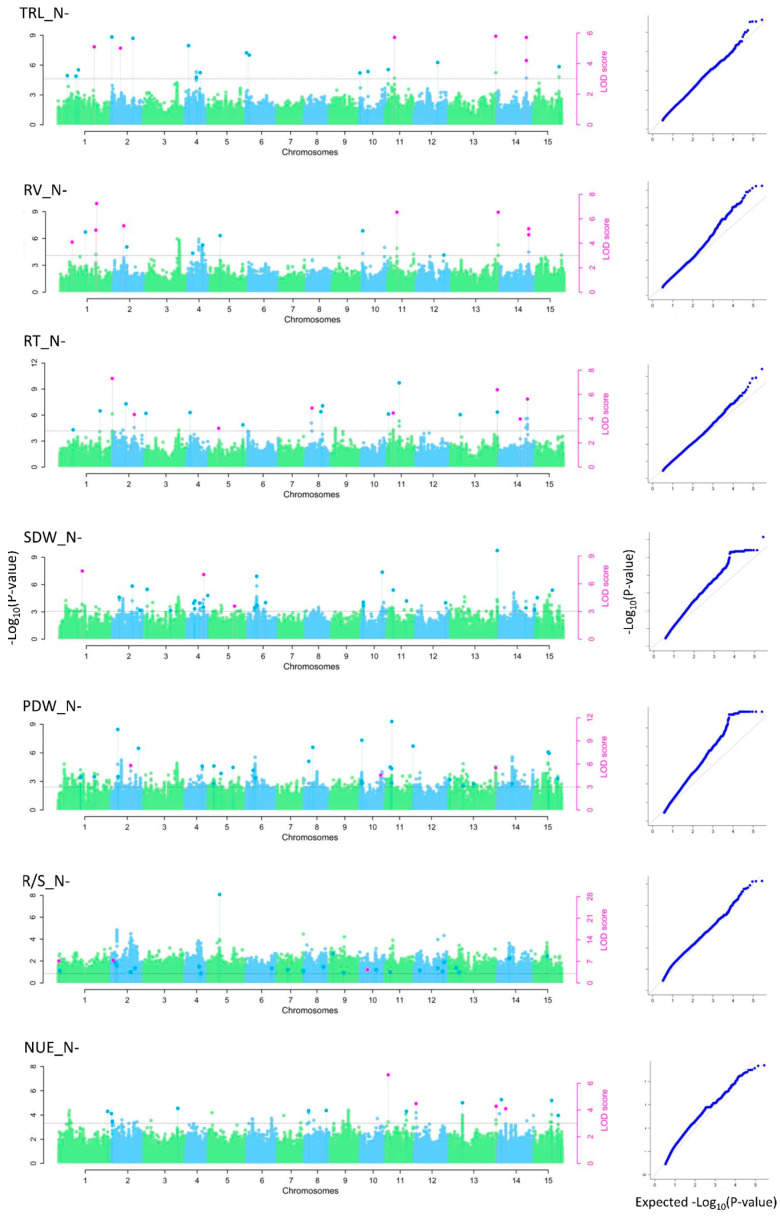
Manhattan (**left panels**) and quantile–quantile (Q-Q) plots (**right panels**) of multi-locus GWAS models of NUE-related traits. QTNs commonly identified by multiple approaches are indicated by the pink dots shown above dotted vertical lines; QTNs identified by a single model are represented by blue dots above vertical lines. The grey horizontal dotted line indicates the genome-wide significance threshold −log_10_(*p*) equivalent to the LOD > 3.0 for ML-GWAS models. TRL_N−: total root length under N−; RV_N−: root volume under N−; RT_N−: number of root tips under N−; SDW_N−: shoot dry weight under N−; PDW_N−: plant dry weight under N−; R/S_N−: root-to-shoot ratio under N−.

The multi-locus methods identified 552 unique QTNs associated with at least one of the 21 traits (Figure 3 and Figure S3). The mrMLM, FASTmrMLM, ISIS EM-BLASSO, FASTmrEMMA and pLARmEB methods detected 64, 231, 91, 32 and 134 of the 552 QTNs, respectively, but some were identified by more than one model. Mann–Whitney non-parametric U tests were conducted on the 552 unique QTNs and the 76 non-significant ones were removed, resulting in a subset of 476 QTNs (*p* < 0.05), which was subsequently merged into 359 QTL based on the LD criteria (*r*^2^ > 0.3) as defined previously. The 359 QTL individually explained 0.11% to 23.07% of the phenotypic variation for the 21 NUE-related traits (Table S3). Of these, 124 were detected by at least two models, and 40 were considered major QTLs as they explained more than 10% of the phenotypic variation (R^2^). One hundred ten QTLs were pleiotropic with at least two traits, which were generally highly correlated. For example, QTL Lu13_19514686 was associated with eight traits, including NUE_N−, RV_N−, PDW_N−, SDW_N−, TRL_N+, RV_N+, RT_N+ and PDW_N+, with correlation coefficients ranging from 0.40 to 0.99 and an R^2^ ranging from 2.41 to 12.85% (Table S3, Figure S1).

**Figure 4:**
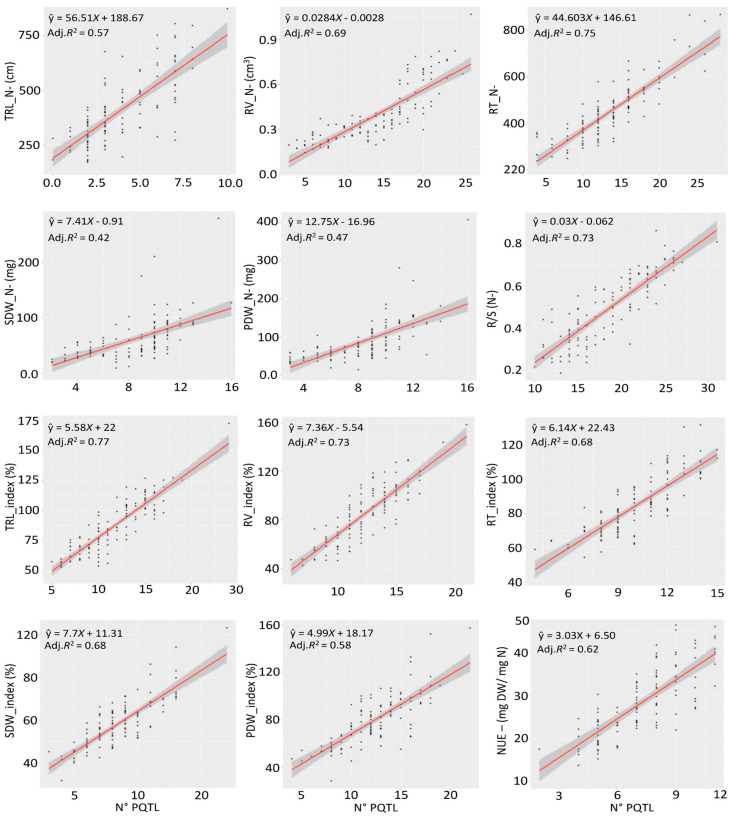
Simple regression analyses of QTL on trait for NUE-related traits and the phenotypic variation explained (adjR^2^) for all detected QTL for each individual trait. TRL_N−: Total root length under N−, RV_N−: Root volume under N−, RT_N−: Number of root tips under N−, SDW_N−: Shoot dry weight under N−, PDW_N−: Plant dry weight under N−, R/S_N−: Root to shoot ratio under N−, TRL_Index: Total root length index, RV_Index: Root volume index, RT_Index: Number of root tips index, SDW_Index: Shoot dry weight index, PDW_Index: Plant dry weight index, NUE_N−: Nitrogen use efficiency under N−.

Simple regression analyses of QTLs for traits revealed moderate to high mean percentage of phenotypic variation explained (adjR^2^), with 0.61, 0.69 and 0.70 for traits under N−, N+ and stability indices, respectively (Figure 4 and Figure S4). QTLs identified under N− explained 42% (SDW_N−) to 75% (RT_N−) of the phenotypic variation, while QTLs detected under N+ condition accounted for 56% (PDW_N+) to 78% (TRL_N+ and R/S_N+). QTLs associated with stability indices explained 58% (PDW_Index) to 77% (TRL_Index) of the traits’ variation (Figure 4 and Figure S4).

The top 10% of accessions (12/123), corresponding to the group of accessions with the highest NUE_N−, and the bottom 10%, corresponding to the group of accessions with the lowest NUE_N−, showed an average NUE_N− of 38.2 (range 26.9 to 46.1) and 19.5 (range 13.7 to 24.7), respectively. Accessions with the highest NUE_N− had, on average, 11 PQTLs (range 10 to 11) associated with this trait, while the genotypes with the lowest NUE_N− had on average four PQTLs (ranging from two to five). The oilseed-type accession O_IRL_C_CN98192 from Ireland had the highest NUE_N− value of 46.1 and contained 331 PQTLs, whereas the Indian accession O_IND_C_CN98982 registered a NUE_N− of 18.1 and had 261 PQTLs out of the 359 identified for the 21 NUE-related traits. O_IRL_C_CN98192 and O_IND_C_CN98982 registered NUE_STI values of 2.55 and 0.33, respectively, suggesting that O_IRL_C_CN98192 has a high NUE and high nitrogen responsive cultivar while O_IND_C_CN98982 has a low NUE and a low nitrogen responsive genotype. These two accessions were selected for root transcriptome analysis.

### 2.4. Differentially Expressed Genes between High and Low NUE Genotypes

A total of 16 libraries, representing two genotypes, two treatments and four biological replicates, were sequenced on an Illumina NovaSeq 6000 platform (San Diego, CA, USA) using the 150 bp paired end read mode, resulting in a total of 411,296,419 million raw reads. After removal of low-quality reads with Trimmomatic v.1.0 402,733,447 high quality reads were found, of which 376,423,963 (92.11%) were mapped to the flax (*L. usitatissimum*) reference genome [48] (Table S4).

Using a principal component analysis (PCA) of gene expression levels, the correlation between biological replicates within the same genotype was greater than the correlation between biological replicates between HN and LN genotypes (Figure S5), which was in agreement with the contrasting N response between genotypes and highlights the accuracy of the four biological replicates.

Root transcriptome differences between HN and LN genotypes after 15 days of N deficiency were determined by performing comparisons between the aligned reads of N− and N+ treatments. A total of 1034 DEGs were identified, of which 108 and 926 were detected in HN and LN genotypes, respectively (Table S5, Figure S6). Among the 108 DEGs observed in HN, 66 and 42 were up and down regulated, respectively. The LN genotype registered 988 DEGs, of which 286 and 702 were up and down regulated, respectively (Table S5, Figure S6).

KEGG enrichment analysis revealed 104 and 223 significantly enriched pathways associated with the up- and down regulated genes, respectively. Among the most enriched pathways, metabolic pathways (map01100), biosynthesis of secondary metabolites (map01110), plant–pathogen interaction (map04626), MAPK signaling plant pathway (map04016), plant hormone signal transduction (map04075), diterpenoid biosynthesis including gibberellin biosynthesis (map00904) and nitrogen metabolism (map00910) were represented more among the up-regulated DEGs (Figure S7a). The KEGG metabolic pathways (map01100), biosynthesis of secondary metabolites (map01110), MAPK signaling plant pathway (map04016), plant–pathogen interaction (map04626), plant hormone signal transduction (map04075), microbial metabolism in diverse environments (map01120) and starch and sucrose metabolism (map00500) were represented more among the down-regulated DEGs (Figure S7b).

Among the DEGs identified, *cytokinin response factor 4* (*CRF4*, *Lus10039324*) and *nitrate regulatory gene2* (*NRG2*, *Lus10029683*) were involved in N signaling (Table 2 and Table S6). DEGs participating in nitrate assimilation, such as *NITRATE TRANSPORTER1/PEPTIDE TRANSPORTER3.1* (*NPF3.1*, *Lus10041466*), *nitrate reductase* (*NIA*, *Lus10035402*) and *SNF1-related protein kinase regulatory subunit beta-2* (*KINB2*, *Lus10038783*), registered altered gene expression patterns in roots in the LN genotype. Genes encoding nitrate transporters like *NITRATE TRANSPORTER1/PEPTIDE TRANSPORTER1.1* (*NPF1.1*/*NRT1.12*, *Lus10014537*), *NITRATE TRANSPORTER1/PEPTIDE TRANSPORTER6.3* (*NPF6.3*/*NRT1.1*, *Lus10032252*) and the *high-affinity nitrate transporter NRT2.1* (*NRT2.1*, *Lus10016120*) were observed in LN roots. Several genes involved in root development, like *NAC021* (*Lus10024908*), *MYB77* (*Lus10010238*) and *WRKY75* (*Lus10011346*) transcription factors, *C-TERMINALLY ENCODED PEPTIDE RECEPTOR 2* (*CEPR2*, *Lus10012461*), *ARABIDOPSIS CRINKLY4* (*ACR4*, *Lus10025455*), *Super Numeric Nodules* (*SUNN*, *Lus10040592*) and *multidrug resistance protein 4* (*MDR4*, *Lus10010012*) were solely transcriptionally altered in the LN accession (Table 2). Various genes involved in amino acid transport also exhibited differential expression patterns, and include *gamma-glutamyl cyclotransferase 2.1* (*GGCT2.1*, *Lus10020181*), *amino acid transporter AAP3* (*AAP3*, *Lus10042740*) and *BASIC AMINO ACID CARRIER 2* (*BAC2*, *Lus10011451*) (Table 2 and Table S6). Notably, 89.5% of the DEGs were down regulated in the root transcriptome of the LN genotype as compared to the HN accession, in agreement with its reduced NUE_STI (Table 2 and Table S6).

### 2.5. Differentially Expressed Candidate Genes at QTLs

**Figure 5:**
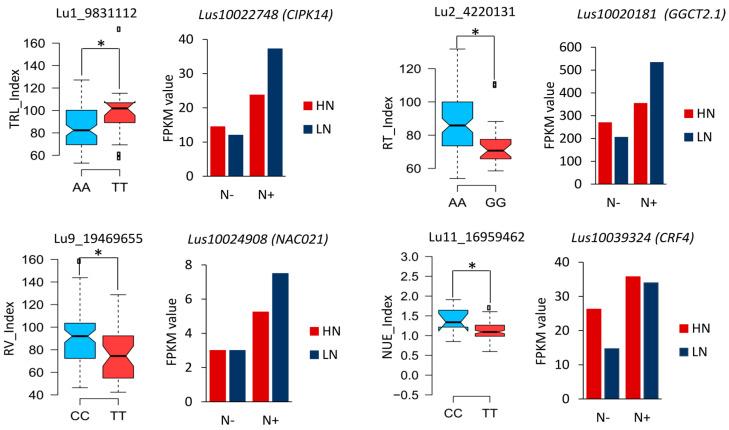
Effects of quantitative trait locus of some NUE-related QTL harboring differential expressed genes (DEGs) identified under contrasting N conditions. Box plots (**left panels**) showing the allelic effect of QTLs associated with NUE-related traits harboring DEGs involved in NUE responses identified in other plant species. Graphics (**right panels**) showing DEGs in QTLs identified under contrasting N conditions in high NUE (HN) and low NUE (LN) flax accessions. FPKM: Fragments per kilobase million. * statistically significant at *p* < 0.05.

The 359 unique QTLs were mined for the identification of differentially expressed candidate genes involved in nitrogen stress responses. The linkage disequilibrium blocks (±100 kb) in the detected QTLs harbored 13,683 genes. Using RNA-seq data from the two NUE contrasting flax accessions, a total of 337 DEGs (32.6%) were found within 169 of these QTL windows (Table S6). The number of DEGs per QTL windows ranged from one to six, with an average of 2 ± 1.04 per QTL. For example, Lu1_9831112 QTL associated with TRL_Index explained 8.7% of the phenotypic variation, and harbored *CBL-interacting serine/threonine-protein kinase 14* (*CIPK14*, *Lus10022748*), the expression of which was increased ~4-fold in LN and nearly doubled in HN under N+ condition compared to N−. (Table 2, Figure 5). Similar trends were observed for Lu2_4220131, a QTL associated with RT_Index that accounted for 11.1% of the phenotypic variation and which carried *gamma-glutamylcyclotransferase 2.1* (*GGCT2.1*, *Lus10020181*), whose expression was increased 2.5-fold in LN under N+ condition compared to N−, whereas in HN the expression patterns were not statistically altered (Figure 5).

The root-development associated DEG NAC021 was located in the pleiotropic QTL Lu9_19469655 associated with R/S_N− and the RV_Index. The expression of NAC021 increased 2.5-fold in LN and nearly doubled in HN under N+ condition compared to N−. Another example of QTL harboring DEGs involved in N stress responses was Lu11_16959462, associated with the NUE_Index, which harbored the N signaling gene CRF4, whose expression patterns were not significantly altered in HN, but increased ~twofold in LN under N+ conditions compared to N−. (Figure 5, Table S6). In addition, QTL Lu4_14298191 linked to R/S_N− harbored the nitrate transporter NPF3.1. The DEG high affinity nitrate transporter 2.5 (NRT2.5) was located in the pleiotropic QTL Lu13_18363934 which is associated with the RT_Index and R/S_N+ (Tables S3 and S6). In general, most of the DEGs in QTLs exhibited altered expression patterns in the LN genotype, but stable expression under N− and N+ conditions in the HN accession.

### 2.6. GWAS-Assisted Genomic Selection

**Figure 6:**
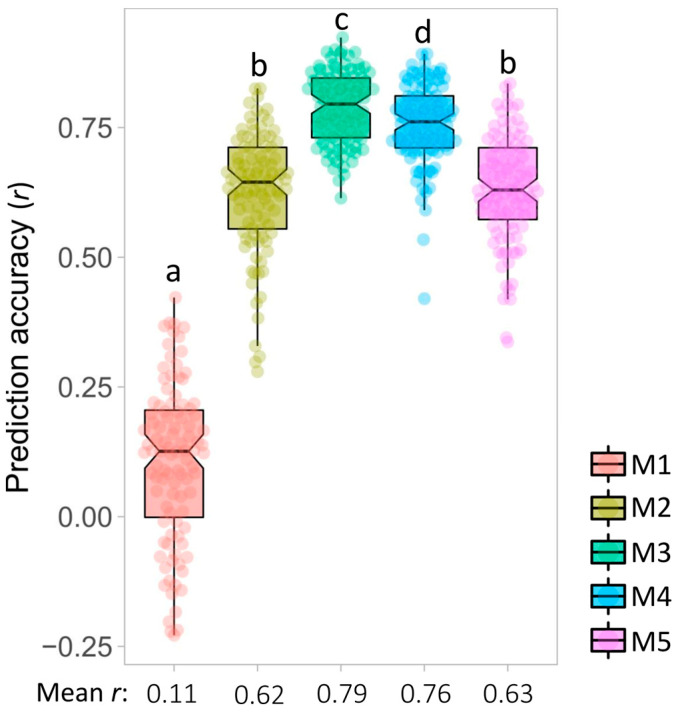
Comparison of genomic prediction accuracy (r) models produced by five different marker sets using the GBLUP statistical model for NUE_STI in flax. M1: marker set 1 using 16,383 genome-wide markers, M2: marker set 2 considering markers associated with all traits under N− condition, M3: marker set 3 including markers associated with NUE_STI, M4: marker set 4 considering markers associated with traits positively correlated with NUE_STI, M5: marker set 5 including markers associated with traits positively correlated with NUE_STI that harbor DEGs. Box plots with the same lowercase letter are not statistically significant at *p* < 0.05.

To identify the marker sets that produce the best prediction accuracy for NUE_STI, the predictive ability of five marker sets (M1 = 16,383 genome-wide markers; M2 = markers associated with all traits under N− condition; M3 = markers associated with NUE_STI; M4 = markers associated with all traits positively correlated with NUE_STI; and M5 = markers associated with all traits positively correlated with NUE_STI that harbored DEGs) were compared using the GS GBLUP model. Analysis of variance in prediction accuracy for the five marker sets showed significant differences (*p* < 0.001). Tukey’s multiple pairwise comparisons showed the highest predictive ability for M3 (r = 0.79), followed by M4 (r = 0.76) (Figure 6). The predictive ability obtained from the other marker sets was significantly lower, with the lowest observed using M1 (r = 0.11) (Figure 6).

## 3. Discussion

Nitrogen plays an important role in root and shoot development and is a critical macronutrient for maximizing yield. Lowering N input and breeding plants with higher NUE are main goals of plant nutrition and sustainable agriculture [9]. Here, we assessed 21 NUE-related traits under N− and N+ conditions for 123 diverse flax accessions during the seedling stage using a multi-omics approach combining GWAS, RNA-seq and GS to understand the flax nitrogen’s response to depleted N conditions and to apply future genomic-assisted breeding strategies.

### 3.1. Phenotypic Variation of Root and Biomass Traits

The concepts of high and low NUE genotypes are widely used in N use efficiency studies. Nonetheless, one must bear in mind that high or low NUE ranking is a relative measurement in comparison to other genotypes within the same experiment. Hence, a high NUE genotype in one study could be a low NUE accession in another depending on the genetic diversity of the panel [42]. In flax, NUE traits and optimum N dosages for efficient flax production have been investigated using, at most, a dozen cultivars [46,47], limiting their broad deployment in breeding programs. Here, an association panel of 123 flax accessions representing the genetic diversity from 28 countries was assembled from the Canadian flax core collection [86], including eighty-three cultivars, twenty-one breeding lines and five landraces that encompass both the oil and fiber morphotypes. The significant and broad variation in root and biomass traits among the genotypes of the association panel agrees with previous studies of similar traits using subsets of the Canadian flax core collection [35,45,87,88]. Thus, this set of flax accessions was considered suitable as a diverse genetic resource for identifying high NUE donor lines for breeding.

Plants grown under N− conditions showed, on average, reduced root and shoot trait values, but increased R/S_N− and NUE_N− than plants grown under N+ conditions, indicating that N-depleted plants can mobilize more carbon to promote root development and, consequently, mine the substrate to acquire N [10,31,42]. In most elite cultivars, increasing the root-to-shoot ratio increases the uptake of N from the deep soil because the longer roots provide optimum nutrient storage in shoots that can be used later at the seed filling stage [89]. The significant and positive correlations between NUE and root traits (r = 0.33 to 0.52) require breeders to consider root traits as selection criteria to improve NUE in flax breeding programs, as observed in maize [10] and rapeseed [31]. Therefore, the selection of optimal root traits, and particularly R/S_N−, at the seedling stage, holds promises to optimize flax NUE.

### 3.2. Genetic Structure and Linkage Disequilibrium

Understanding the genetic structure and extent of LD in germplasm resources is crucial for conducting reliable GWAS. Population structure and cryptic relatedness (kinship) are the main factors influencing the number of false marker–trait associations, while LD is the main factor influencing marker density requirement and mapping resolution in association studies [90]. Here, we observed weak kinship relationships among accessions and rapid LD decay for most of the chromosomes. The 123 flax accessions are expected to contain ample allelic diversity, as suggested by the generally small LD blocks for the 15 chromosomes, thereby minimizing the occurrence of false positives and facilitating the search of N stress candidate genes through efficiently narrowing the putative QTL regions.

### 3.3. ML-GWAS of 21 NUE-Related Traits in Flax

Each step contributing to NUE, such as uptake, translocation, assimilation and remobilization of N, is a complex process controlled by many molecular, physiological and metabolic root- and shoot-related traits that may exert independent or synergistic effects on NUE, complicating the comprehensive dissection of its genetic architecture. GWAS has become a widely used method for the genetic dissection of complex traits like NUE-related traits in *Triticum aestivum* (wheat) [28,91], *Zea mays* (maize) [29,92], *Brassica napus* (rapeseed) [7,31] and *Hordeum vulgare* (barley) [93]. In flax, GWAS have been conducted for agronomic, seed quality and disease resistance traits [94,95,96,97], as well as for drought-related traits and early root and shoot development [35,87,88,98].

Using six ML-GWAS models, 359 unique QTLs, distributed over all 15 chromosomes, were detected for 21 NUE-related traits. Multi-locus models consider multiple QTLs in the model and treat them as random effect, which assumes that multiple QTLs control the phenotype [99]. Because all the potential QTLs for the quantitative traits are fitted to a single linear model and their effects are estimated and tested simultaneously, the classical stringent Bonferroni correction is unwarranted because the detection of many small effect QTLs is key to detect the full breadth of the QTLs underlying complex traits [95]. To maximize the robustness and reliability of the significant marker-trait associations, the Mann–Whitney non-parametric U tests were carried out to remove false positive QTLs that did not produce a significant allelic effect on the traits. In flax, this approach has been applied for flowering time [100], root and shoot development [87,88,98], drought-related traits [35,87] and disease resistance traits [95,101], totaling 1478 QTLs identified, allowing for their application in GWAS-assisted genomic selection strategies [41,95,101].

The high number of pleiotropic QTLs (n = 110) indicates the close genetic relationships between most of the 21 traits assessed (Table 2). Importantly, NUE and root traits were co-located at fifteen pleiotropic QTLs, whereas NUE and shoot traits shared the same genomic regions at only three QTLs. The important genetic associations between root traits and NUE are not only seen in the seedling stage, but also at the reproductive stage, as previously reported in other crops [10,31,102]. Accordingly, the significant relationships found between root QTLs and yield under water stress in flax [35] and phosphorus deficiency in maize [103] further support the essential role of root traits on adaptation of crops to abiotic stresses.

### 3.4. Differentially Expressed Genes between High and Low NUE Genotypes

The RNA-seq approach has been applied in many crops for multiple traits to identify gene networks [104]. Here, we identified 1034 DEGs in the roots of two flax genotypes with contrasting NUE, where most genes showed altered patterns of expression in the low NUE (LN) genotype. KEGG pathway analysis showed that DEGs involved in metabolic pathways, the biosynthesis of secondary metabolites, plant–pathogen interaction and some genes involved in N metabolism and phenylpropanoid biosynthesis were significantly enriched. Key metabolic processes such as energy production, N assimilation and root development, among others, were repressed in LN, while they remained unaltered in HN, indicating its greater potential to cope with N depleted environments. Similar KEGG pathway results in roots submitted to N starved conditions have been reported in rice [105] and cotton [106].

Our study provides the first insight into N stress-responsive genes hypothesized to have important roles in N stress adaptation in flax. The distinct transcriptional response observed in both flax accessions in response to starved N condition may serve as a roadmap to understanding the genetic factors and their interactions in governing NUE in flax.

Through gene annotation and literature reports, at least fifteen identified DEGs are involved in N metabolism, of which ten were downregulated in LN. For instance, a mutation in *AtNRG2*, the *Arabidopsis* ortholog of flax *Lus10029683*, disrupted the induction of nitrate-responsive genes after nitrate treatment, where the nitrate content in roots was lower in the mutants than in the wild type [50]. The reduced nitrate content in roots may have resulted from a reduced expression of *NPF6.3/NRT1.1*, the *Arabidopsis* ortholog of flax *Lus10032252. NPF6.3/NRT1.1* is a bidirectional transporter involved in root-to-shoot nitrate translocation [58], which was also downregulated in LN. Root traits play a pivotal role in response to N stress and superior NUE in plants. Here, we identified seventeen DEGs that modulate root development, of which nine were involved in lateral root development. In general, N deficiency promotes root growth including root length, diameter and volume, and lateral root length depending on soil environment and N distribution [7,27,28,29,31]. *Lus10024908*, the ortholog of *Arabidopsis NAC021* mediates auxin signaling to promote lateral root development, and its overexpression results in a higher number and longer lateral roots [69]. Another interesting DEG involved in lateral root development was *Lus10040592*. Its ortholog in *Arabidopsis* is *SUNN*, which controls lateral root density in response to N concentration through the modulation of shoot-to-root auxin transport [75]. Lateral roots are organs that are under the control of nutrient supply such as N or phosphate, and modification of their architecture is a key mechanism underlying plant response to nutrient depleted soil conditions [65,75]. For example, *Lus10011346*, the ortholog of *WRKY75* transcription factor, mediates phosphate acquisition and lateral root development in *Arabidopsis* [65]. Most of the DEGs involved in root development were downregulated in the LN genotype, in agreement with its 36.3, 46.8 and 36.1% reductions in TRL, RV and RT, respectively, under N− compared to the HN genotype. Taken together, the root transcriptome analysis provided a valuable catalog of DEGs, which further support the negative effects of N stress on biomass production, root architecture, N content in roots and shoots, energy production and ultimately NUE, as witnessed in *Brassica juncea* [107], *Solanum tuberosum* [104] and *Brassica napus* [31].

### 3.5. Differentially Expressed Genes at QTLs

Omics research is now shifting from single-omics to large-scale multi-omics approaches [108]. Through this approach, an understanding of the fundamental biological processes can be achieved for a more accurate prediction of the response variable, and we can gain further insights into the mechanistic aspects of the system [108]. Through the multi-omics approach, researchers can obtain a deeper understanding of the fundamental biological processes, attain more accurate predictions for the response variables and gain further insights into the mechanistic aspects of the system [108]. Combining GWAS with RNA-seq improves the accuracy of candidate gene selection [109]. For example, eight salt stress-related candidate genes were identified by a combination of GWAS and transcriptome analysis in Medicago sativa (lucerne) [110]. Through the integration of GWAS and RNA-seq, fourteen candidate genes responsive to drought stress were identified in *Helianthus annuus* (common sunflower) [109]. Similarly, combining QTL mapping, GWAS and transcriptome analysis enabled the identification of genes involved in high-temperature stress tolerance in *Oryza sativa* (rice) [111]. Here, the root RNA-seq analysis allowed us to locate 337 DEGs in 169 QTLs (47.1%), to identify DEGs in NUE-related QTLs and to confirm the robustness of the mathematical algorithms upon which ML-GWAS analyses rely.

Both carbon (C) and N nutrients are essential for various cellular functions, and therefore adequate supply of these two nutrients is critical for plant growth and stress response. *CBL-interacting serine/threonine-protein kinase 14* (*CIPK14*, *Lus10022748*) has been shown to coordinate the responses to oxygen and sugar deficiencies in rice and coordinate the C and N signaling pathways in response to the relative C/N status in rice seedling roots [51]. *CIPK14*, located in QTL Lu1_9831112 and associated with TRL_Index, was upregulated in the LN genotype. Similarly, *Gamma-glutamylcyclotransferase 2.1* (*GGCT2.1*, *Lus10020181*) catalyzes the formation of 5-oxoproline from gamma-glutamyl dipeptides and plays a significant role in glutathione (GSH) homeostasis. In *Arabidopsis*, *GGCT2.1* mobilizes L-cysteine from glutathione when there is insufficient sulfate for de novo L-cysteine synthesis, and under sulfur-starvation, induces changes in the root system architecture through activity of the gamma–glutamyl cycle in the primary root tip [79]. In our study, QTL Lu2_4220131 was found to be associated with the RT_Index, and carried the up-regulated *GGCT2.1* gene, which was up-regulated under N−, suggesting that both sulfur and N starvation could initiate the same glutathione degradation mechanism to produce the component amino acids L-glutamate, L-cysteine and L-glycine. More than 50 distinct amino acid transporter genes have been identified in the *Arabidopsis* genome, indicating that the movement of amino acids across membranes is a highly complex process in plants. The *Arabidopsis* ortholog *cationic amino acid transporter 8* (*CAT8*, *Lus10005574*) located in QTL Lu14_17132252 (R/S_N+) is involved in the movement of the cationic neutral or acidic amino acids and is preferentially expressed in young and rapidly dividing tissues such as young leaves and root apical meristem [78]. Thus, *CAT8* may be involved in allocation of the amino acids’ glutamine and glutamic acid to root and shoot meristems as precursors for the synthesis of other amino acids under N− [78].

In plants that have been deprived of nitrate for a significant length of time, a constitutive high-affinity nitrate transport system was shown to be responsible for initial nitrate uptake [59]. *Arabidopsis high affinity nitrate transporter 2.5* (*NRT2.5*, *Lus10030902*) included within the LD block of QTL Lu13_18439316 (RT_Index, R/S_N+) is predominantly expressed in roots of nitrate-deprived plants as a 150 kDa molecular complex with NRT3.1. This complex accounts for 63% of the constitutive high-affinity nitrate transport system influx and is the major contributor to nitrate absorption [59].

Root development and root architecture plasticity are pivotal processes in N stress responses. In Arabidopsis, ethylene is biosynthesized from S-adenosyl-L-methionine through *1-aminocyclopropane-1-carboxylate oxidase 1* (*ACO1*, *Lus10008564*). In flax, *ACO1*, located in QTL Lu11_14898826 (NUE_N−), could play a regulatory role in lateral root development, as in *Arabidopsis* where *aco1* mutants showed reduced ethylene production in root tips compared to wild-type and displayed altered lateral root formation [62]. *LRR receptor-like serine/threonine-protein kinase GSO1* (*GSO1*, *Lus10036251*) in coordination with *GSO2*, regulates seedling root growth through control of cell division and cell fate specification [63]. *GSO1*, a DEG located in QTL Lu12_1386203 and associated with RT_N+ and R/S_N−, is a prime candidate considering its role in root development.

Together, the DEGs identified at QTLs and their important and well documented roles in N metabolism, root development, energy production, amino acid transport/catabolism and diverse abiotic stresses demonstrate the efficacy of combining omics tools for rapid identification of candidate genes controlling complex traits like NUE and other related abiotic stresses [31,35,109].

### 3.6. GWAS-Assisted Genomic Selection

Genomic selection (GS) is a promising approach used in crop breeding programs, particularly for improving complex traits. In GS, genome-wide markers are used to predict the genomic-estimated breeding values (GEBVs) of individuals by capturing the benefits of both major- and minor-effect QTLs [36]. By replacing the phenotypic selection with the GEBV, the genetic gain for each unit cycle can be increased [112]. GS reduced the selection cycle length of maize, *Arabidopsis* and barley breeding programs compared to phenotypic selection that otherwise could take several cycles of extensive phenotyping to develop reliable phenotypic data [113]. However, performing GS using a large number of markers is challenging, due to the curse of dimensionality as well as multicollinearity arising from LD between markers [114]. In addition, molecular markers are major factors affecting both genomic prediction accuracy (r) and the cost of GS [41]. Previous studies have indicated that the use of QTLs as markers in GS significantly increases prediction accuracy compared with genome-wide random markers [41,95,101]. This approach, also known as GWAS-assisted GS, produced a superior prediction accuracy of 0.92 when 500 QTLs associated with pasmo resistance were used compared to an accuracy of 0.67 when 52,347 random SNPs were employed in the GS models in flax [95]. Similarly, the GWAS-assisted GS strategy outperformed the prediction accuracies obtained with 17,277 genome-wide SNPs for seven breeding selection traits in flax including yield, days to maturity, iodine value, seed protein content, oil content, linoleic acid and linolenic acid contents [41]. Here, we compared GBLUP GS models to predict NUE_STI using five marker sets including a random genome-wide marker set (M1 = 16,383 SNPs) and four GWAS-QTL-based marker sets (M2-M5). Our results confirmed the superiority of all GWAS-based marker sets for GS in NUE_STI (r = 0.62 to 0.79) over the random genome-wide marker set (r = 0.11), as previously reported in flax [41,95,101] and maize [115]. Interestingly, the highest prediction accuracy for NUE_STI (r = 0.79) was observed when the smallest marker dataset of only 16 trait-specific QTLs was used. Because pleiotropy suggests that different traits might be genetically controlled by the same or tightly linked genes/QTLs, we hypothesized that traits correlated with NUE_STI are genetically controlled by pleiotropic QTLs that can be used as markers in GS to improve prediction accuracy. Our results rejected the hypothesis, indicating that QTLs from correlated traits did not overcome GS accuracy as compared with trait-specific QTLs, but was the second-best model. This outcome might be caused by QTLs’ redundancy or background noise as observed for seven breeding selection traits in flax [41] and should be evaluated on a case-by-case basis, depending on the trait and its correlated contributing traits. Hence, our results suggest that trait-specific QTLs not only significantly improved prediction accuracy, but also reduced the number of markers, which in turn would decrease genotyping cost in practical breeding programs [41,101]. However, as we used as validating population different partitioning of the same 123 accessions, a potential confounding effect was observed as artificial inflation of the predictive ability might occur for the QTL data sets. As a recommendation to minimize artificial inflation, the best GS model should be tested in an independent set of genotypes [116].

Another important factor determining higher prediction accuracy is the size of the training population and its genetic diversity. In flax, using different training population sizes derived from the Canadian flax core collection (n = 370), the prediction accuracies for pasmo resistance ranged from 0.8 with 50 genotypes to 0.9 with 185 accessions, whereas smaller accuracy gains up to 0.93 were obtained between 200 and 370 individuals [95]. Here, we used a training population of 123 diverse individuals, which, based on previous reports, is expected to provide sufficient genetic diversity and, therefore, abundant favorable QTL alleles for NUE-related traits useful to construct robust GS models [35,87,95]. Despite the prediction accuracy for NUE_STI being the highest when the GBLUP model was performed with the 16 trait-specific QTLs marker set, the evaluation of additional parametric and non-parametric GS models could improve r values for NUE_STI, as observed for maize [115] and wheat [116]. However, the highest prediction accuracies not only depend on the GS model, but also on the genetic architecture of the target trait, the extent of LD, and the genetic diversity of the training population, factors that should be addressed before implementing practical breeding programs.

## 4. Materials and Methods

### 4.1. Plant Materials

An association panel consisted of 123 flax accessions and representing the genetic diversity from 28 countries was assembled from the Canadian flax core collection [86]. The plant materials include 83 cultivars, 21 breeding lines, 5 landraces and 14 accessions of unknown improvement status grouped into 88 oilseed and 35 fiber morphotypes (Table S1).

### 4.2. Plant Growth Conditions and Phenotyping

Seeds from each of the 123 flax accessions were germinated in Petri dishes on filter paper wet with distilled water. After seven days, two uniform seedlings were transplanted into pots (648 cm^3^) filled with sterile silver sand. Seedlings were watered daily with 30 mL of modified Hoagland’s nutrient solution. Briefly, the nutrient solution contained 2.5 mM K_2_SO_4_, 2 mM MgSO_4_, 1 mM KH_2_PO_4_, 1 mL L^−1^ Hoagland micronutrients and 2 mL L^−1^ FeEDTA solution [12]. Two treatments were evaluated with nitrate added to the solution in the form of 500 mM Ca(NO_3_)_2_, to obtain NO_3_^−^ concentrations of 2.5 mM, referred to as N+ or (0 mM N) referred to as N−. To adjust Ca^2+^ concentration to the same values in both N treatments, 0.9 mM CaSO_4_ was added to the N− treatment. Pots were moved to a greenhouse facility maintained at 18–25 °C, with an 18 h day/6 h night photoperiod and 30–50% relative humidity. The photosynthetically active radiation (PAR) was approximately 400 µmol m^−2^ s^−1^. The experiment was laid out as a completely randomized design with three biological replicates, totaling six plants per genotype. After 14 days, pots were immersed in water to loosen the soil and the roots were released. The seedlings from each N treatment and genotype were collected and cut into root and shoot sections. The root system of each plant was imaged using a calibrated optical scanner LA2400 (Epson 11000XL, Long Beach, CA, USA). Root images were analyzed using the WinRHIZO software v.2.0 (Regent Instruments, Montreal, QC, Canada), and the measurement data obtained was used to calculate the following root traits: (i) total root length (TRL; cm), (ii) root volume (RV; cm^3^) and (iii) number of root tips (RT). Thereafter, plant tissues were placed in an oven at 60 °C for two days, after which plant dry weight (PDW), root dry weight (RDW), shoot dry weight (SDW) were determined, and root/shoot ratio was calculated. Shoot and root N content were determined using the Dumas combustion method in a Gerhardt, DUMATHERM^®^ N Pro analyzer as described by Muñoz-Huerta [117].

### 4.3. Phenotypic Data Analysis

Statistical differences between treatments, genotypes and genotype x treatment interactions for shoot and root traits were analyzed using a Restricted Maximum Likelihood (REML) analysis implemented in GenStat v.18 [118] with *p* < 0.05 as threshold. Best linear unbiased estimates (BLUEs) were obtained for each trait under both N treatments and used to calculate the trait stability indices using the ratio of the trait under N− to the trait under N+ [119]. NUE values were calculated using the following formula: plant dry weight (mg)/Plant N content (mg). NUE stress tolerance index (NUE_STI) was estimated according to Fernandez [120]. All traits were used as input for the multi-locus GWAS analyses. Pearson’s correlation analyses were conducted using the R package “ggplot2”v.3.4.4 [121] to determine the correlations between the 21 NUE-related traits and their distributions under N− and N+ conditions.

### 4.4. Genotyping, Genetic Structure and Linkage Disequilibrium

SNP data was generated for the entire flax core collection (n = 407) by resequencing all the accessions using Illumina HiSeq 2000 platform in a 100 bp paired-end mode at an average genome coverage of 17× and generating a data set of 570,443 high-quality SNPs with a call rate > 80%. From this core data set, SNP information was extracted for the above mentioned 123 accessions and further filtered at a call rate > 95% and a minor allele frequency (MAF) > 0.05, and the resulting 272,944 SNP dataset was used for this study.

The genetic structure of the 123 accessions was evaluated in STRUCTURE v.2.3.4 [122] using a subset of 5305 SNPs generated from the 272,944 SNP dataset at a call rate > 98% and a MAF > 0.05 and that were found evenly distributed across the 15 pseudomolecules of flax [48]. The number of sub-groups was determined using the web-based software Structure Harvester v.0.6.94 (http://taylor0.biology.ucla.edu/structureHarvester/, (accessed on 14 July 2023)), which is based on the Evanno method [123]. Neighbor-joining (NJ) phylogenetic, principal component (PC) and kinship analyses were performed based on the 272,944 SNPs using TASSEL v 5.2.31 [124].

Genome-wide and chromosome-specific linkage disequilibrium (LD) were estimated using the squared correlations of allele frequency (*r*^2^) in TASSEL v.5.2.31 [124] with a sliding window size of 50. LD decay was estimated as previously described by Soto-Cerda [94].

### 4.5. Multi-Locus Genome-Wide Association Analyses

To identify the genetic loci underlying NUE-related traits, the ML-GWAS methods FASTmrEMMA [125], FASTmrMLM [126], ISIS EM-BLASSO [127], mrMLM [128], pKWmEB [129] and pLARmEB [130] included in the R package multi-locus random-SNP-effect mixed linear model (mrMLM v. 4.0.2) [131], were computed using the default parameters. A logarithm of the odds (LOD) score > 3.0 was set as threshold to detect robust marker-trait associations, which increases the QTN detection power compared to single-locus GWAS while reducing type I errors [125,126,127,128,129,130]. The ML-GWAS outcomes were summarized and displayed using Manhattan plots, and their ability to minimize false-positive associations was tested with quantile–quantile (Q-Q) plots. The mrMLM v. 4.0.2 package constructs the Manhattan and Q-Q plots using the −log_10_(*p*) median among the −log_10_(*p*) values of each marker obtained from the mrMLM, FASTmrMLM, FASTmrEMMA and pKWmEB models. Irrespective of the −log_10_(*p*) median, only significant markers (LOD > 3.0) are therefore shown in the Manhattan plots above dotted vertical lines [131].

Significant QTNs detected by the ML-GWAS analyses were further filtered using the Mann–Whitney non-parametric U test (*p* < 0.05). QTNs with non-significant allele effect were considered false positives and were removed. The QTNs with significant allele effect were grouped into QTL blocks if they belonged to a physical distance corresponding to 75% of the maximum LD decay. As such, neighboring QTNs within QTL blocks had a high probability of remaining linked across generations [132]. For each QTL block, the QTN with the largest % of phenotypic variations explained (R^2^) value was selected as the representative QTN for the QTL block. Boxplots to visualize QTL allele effect were made using the R package “ggplot2” [121]. Adjusted R^2^ (adjR^2^) values for all QTL associated with NUE-related traits were calculated based on simple regressions of QTL on traits because the adjR^2^ values represent the proportion of the total variation of traits explained by the QTL [95].

The simple regression analyses were conducted using Blue Sky statistics (https://www.blueskystatistics.com/ (accessed on 10 May 2023)).

### 4.6. Transcriptome Sequencing and Analysis

From the N stress experiment described above, two contrasting flax accessions O_IRL_C_CN98192 and O_IND_C_CN98982, referred to as high NUE (HN) and low NUE (LN) hereafter, respectively, were selected and submitted again to the same N stress experiment using the modified Hoagland’s nutrient solution as described above. Root samples were collected 14 days after transplanting and immediately frozen in liquid nitrogen. The experiment included four biological replicates per genotype and treatment (N− and N+), for a total of 16 experimental units. Total RNA was extracted from the root tissue of each of the 16 biological sample units using the Spectrum Plant Total RNA kit (Sigma-Aldrich, St. Louis, MO, USA). RNA was DNase-treated, visualized on an agarose gel, quantified using a Nanodrop (Thermo Scientific, Madison, WI, USA) and RNA QC for all samples was ensured by RNA integrity number (RIN) ≥ 7.0 using a 2100 Bioanalyzer (Agilent Technologies, Santa Clara, CA, USA). The 16 RNA libraries were constructed by outsourcing to Novogene Corporation Inc. (Beijing, China) and were sequenced in paired-end mode using a NovaSeq 6000 platform (Illumina, Inc., San Diego, CA, USA) through 150 cycles. The sequencing results in FASTQ format were processed with Trimmomatic v.1 [133] which involved the removal of bases with a q-score below 15 from both their 5′ and 3′ ends, while retaining sequences that were longer than 70 base pairs. The high-quality reads were mapped to the flax reference genome [48] using HISAT2 v2.2.1 [134] with default parameters The alignment bam files were processed and sorted with samtools v1.3.1 [135] and the abundance of genomic features was computed using featureCounts v2.0.1 [136]. The statistical differences in gene expression were assessed with the R package DESeq2 [137] with the threshold set as |log2(Fold change)| ≥ 1 and false discovery rate (FDR)-adjusted *p* value < 0.05.

Functional annotation of the DEGs was carried out against the curated KEGG GENES database using the KEGG Automatic Annotation Server (KAAS, https://www.genome.jp/kegg/ (accessed on 12 January 2023)). KEGG Orthologs (KO) were assigned using the KofamKOALA software [138].

### 4.7. Cross-Referencing of Differentially Expressed Genes and QTL-Associated Candidate Genes

GWAS-detected QTL were scanned within an estimated average genome-wide LD decay range of 100 kb (Figure 2d) for the identification of candidate genes mined from the flax reference genome [48] using the Jbrowse feature of Phytozome v.13.1 (http://phytozome.jgi.doe.gov/pz/portal.html (accessed on 13 January 2023)).

Candidate genes identified near the QTL were further analyzed and cross-referenced with the root RNA-seq transcriptomic data from the two contrasting flax accessions HN and LN for their involvement in nitrogen stress responses. Only DEGs with a fold change ≥ 1 and an FDR-adjusted *p* value < 0.05 were considered for cross-referencing with candidate genes associated with the QTL. To determine the biological significance of the DEGs, *L. usitatissimum* genes were annotated by BLASTP against the SwissProt database using the threshold criteria of identity ≥ 50%, sequence coverage ≥ 70% and E-value ≤ 1 × e^−10^. The functional role of DEGs in nitrogen stress responses were further examined by searching literature reports for their functional characterization in other plant species.

### 4.8. GWAS-Assisted Genomic Selection

Markers (QTLs) obtained from the ML-GWAS were used to predict the GEBV of NUE_STI, a trait that allows the identification of genotypes with high NUE and high N responses. Five marker sets were tested as GS input. Marker set 1 (M1) was constructed using 16,383 random genome-wide markers; marker set 2 (M2) considered the markers identified by GWAS to be associated with all traits under the N− conditions (n = 120); marker set 3 (M3) included only the markers associated with NUE_STI (n = 16); marker set 4 (M4) was constructed with the markers associated with all traits positively correlated with NUE_STI (n = 281); and marker set 5 (M5) was assembled using the markers associated with all traits positively correlated with NUE_STI that harbored DEGs (n = 127). The goal was to determine the best dataset to obtain the highest prediction accuracy for NUE_STI, considering QTL identified for NUE_STI per se, and those of traits correlated with it [139].

The genomic BLUP (GBLUP) statistical model, implemented in the R package BGLR (https://r-forge.r-project.org/projects/bglr/, accessed on 12 January 2023) [140], was used to evaluate prediction accuracy of the five marker sets because GBLUP’s superior prediction ability for highly polygenic traits has been demonstrated in flax [41,101]. The computation methodology of GBLUP has been previously described in more detail by De Los Campos [141]. The marker sets were formatted as “1” for the positive effect allele of a QTL and “−1” for the alternative allele [95].

For GS model validation, a fivefold random cross-validation strategy was applied [95]. The 123 accessions were randomly partitioned into five subsets. For a given partition, each subset was used as test data, whereas the remaining four subsets were used as a training dataset. This partitioning was repeated 100 times. The accuracy of GS (r) was computed using the Pearson’s correlation coefficient between the genetic values predicted by GS and the observed phenotypic values. To compare the different marker sets, an analysis of variance (ANOVA) with Tukey’s multiple pairwise comparisons was conducted to determine the statistical significance of differences in genomic prediction (r) using GenStat v18 [118] with *p* < 0.05. Boxplots for each marker set were made using the R package “ggplot2” [121].

## 5. Conclusions

The 123 flax accessions exhibited abundant genetic diversity for NUE-related traits, in line with their diverse geographic origin, breeding status and plant morphotype, resulting in the identification of 359 NUE-related trait QTLs. This study reaffirmed the polygenic nature of NUE and its related traits which are controlled by many small and medium effect QTLs in flax. Root RNA-seq analysis identified important candidate genes involved in root development and N metabolism, among which 337 candidates were found at 169 QTLs. GWAS-assisted GS strategy produced superior genomic prediction accuracies compared to genome-wide markers for NUE_STI, and the GS model based on trait-specific QTLs had the best predictive ability (r = 0.79). The use of GWAS-derived QTL associated with a target trait is recommended because it is the most accurate, cost-effective and computationally advantageous for NUE and other quantitative traits in flax.

## Figures and Tables

**Table 1:** Phenotypic variation in 21 NUE-related traits under optimum and depleted N conditions in flax.

Trait	Mean	Standard Deviation	Range	C.V. (%)
Total root length N+ (cm)	469.2	137.7	168.6–882.0	29.2
Total root length N− (cm)	407.2	156.8	171.0–870.9	38.3
Root volume N+ (cm^3^)	0.464	0.181	0.161–1.056	38.7
Root volume N− (cm^3^)	0.389	0.188	0.143–1.069	48.1
Number of root tips N+	533.7	120.7	292.4–1060.4	22.5
Number of root tips N−	447.8	125.1	236.6–864.5	27.8
Plant dry weight N+ (mg)	105.7	52.9	11.8–390.9	49.8
Plant dry weight N− (mg)	86.4	53.3	6.8–405.8	61.5
Shoot dry weight N+ (mg)	77.1	41.3	9.1–290.6	53.3
Shoot dry weight N− (mg)	59.9	38.5	4.6–280.0	64.0
Root/shoot N+	0.407	0.116	0.215–0.784	28.3
Root/shoot N−	0.490	0.158	0.188–0.862	32.0
TRL_Index (%)	87.3	21.6	53.1–172.4	24.7
RV_Index (%)	85.1	23.7	42.4–158.4	27.7
RT_Index (%)	84.6	16.9	53.9–131.8	19.9
PDW_Index (%)	80.1	21.1	28.7–156.9	26.2
SDW_Index (%)	76.1	22.9	22.9–166.7	30.0
NUE N+	25.6	7.2	10.4–37.2	28.0
NUE N−	28.3	7.8	13.7–46.5	27.5
NUE_Index (%)	114.6	25.0	59.7–204.7	21.7
NUE_STI	1.17	0.571	0.23–2.55	48.5

TRL: total root length; RV: root volume; RT: number of root tips; PDW: plant dry weight; SDW: shoot dry weight; NUE: nitrogen use efficiency; STI: stress tolerance index; C.V.: coefficient of variation.

**Table 2:** Differentially expressed genes involved in NUE-related traits in flax functionally that have been validated in other plant studies.

Flax DEG	Gene Name	Protein Name	QTL	Trait	Function	Up/Down (Log_2_FC)	Reference
*Lus10027573*	*CRF4*	Ethylene-responsive transcription factor CRF4	Not in QTL	Unknown	N signaling	Up LN (1.20)	[49]
*Lus10029683*	*NRG2*	Nitrate regulatory gene2 protein	Lu9_18443162	RV_Index	N signaling	Down LN (−1.33)	[50]
*Lus10019292*	*LBD38*	LOB domain-containing protein 38	Not in QTL	Unknown	NO_3_^−^ response regulation	Up LN (1.27)	[51]
*Lus10038783*	*KINB2*	SNF1-related protein kinase regulatory subunit beta-2	Lu11_1841121	NUE_N−, NUE_N+, NUE_STI	NO_3_^−^ assimilation	Down LN (−1.32)	[52]
*Lus10041466*	*NPF3.1*	Protein NRT1/PTR FAMILY 3.1	Lu4_14298191	R/S_N−	NO_3_^−^ assimilation	Up LN (1.49)	[53]
*Lus10035402*	*NIA*	Nitrate reductase [NADH]	Not in QTL	Unknown	NO_3_^−^ assimilation	Down LN (−1.26)	[54]
*Lus10027270*	*NR*	Nitrate reductase [NADH]	Not in QTL	Unknown	NO_3_^−^ assimilation	Down LN (−1.46)	[55]
*Lus10004037*	*GS1-2*	Glutamine synthetase cytosolic isozyme 2	Not in QTL	Unknown	NH_4_^+^ assimilation	Down LN (−1.37), Down HN (−1.03)	[56]
*Lus10029256*	*CEP14*	Precursor of CEP14	Not in QTL	Unknown	NH_4_^+^ assimilation, root development	Down LN (−1.16)	[57]
*Lus10016120*	*NRT2.1*	High-affinity nitrate transporter 2.1	Not in QTL	Unknown	NO_3_^−^ transporter	Down LN (−1.17)	[58]
*Lus10030902*	*NRT2.5*	High affinity nitrate transporter 2.5	Lu13_18363934	RT_Index, R/S_N+	NO_3_^−^ transporter	Down LN (−2.38), Down HN (−1.83)	[59]
*Lus10014537*	*NPF1.1*	Protein NRT1/PTR FAMILY 1.1	Not in QTL	Unknown	NO_3_^−^ transporter	Up LN (1.12)	[18]
*Lus10032876*	*NPF4.6*	Protein NRT1/PTR FAMILY 4.6	Not in QTL	Unknown	NO_3_^−^ transporter	Down LN (−1.06)	[60]
*Lus10032252*	*NPF6.3*	Protein NRT1/PTR FAMILY 6.3	Not in QTL	Unknown	NO_3_^−^ transporter	Down LN (−1.21)	[58]
*Lus10004760*	*AMT1-2*	Ammonium transporter 1 member 2	Lu14_9866805	RV_N−	NH_4_^+^ transporter	Down LN (−3.14), Down HN (−2.20)	[61]
*Lus10008564*	*ACO1*	1-aminocyclopropane-1-carboxylate oxidase 1	Lu11_14898826	NUE_N−	Root development	Up LN (1.04)	[62]
*Lus10036251*	*GSO1*	LRR receptor-like serine/threonine-protein kinase GSO1	Lu12_1386203	RT_N+, R/S_N−	Root development	Up LN (1.91)	[63]
*Lus10016554*	*FLA4*	Fasciclin-like arabinogalactan protein 4	Lu12_4334157	PDW_N+	Root development	Down LN (−1.02)	[64]
*Lus10011346*	*WRKY75*	Probable WRKY transcription factor 75	Lu13_19862281	TRL_Index	Root development	Up LN (1.46)	[65]
*Lus10042154*	*FH8*	Formin-like protein 8	Lu14_15927781	RV_N+	Root development	Up LN (1.53)	[66]
*Lus10021428*	*MYB36*	Transcription factor MYB36	Lu14_3980668	R/S_N−	Root development	Down LN (−1.02)	[67]
*Lus10035126*	*RPK2*	LRR receptor-like serine/threonine-protein kinase RPK2	Lu2_21668160	PDW_N+	Root development	Down LN (−1.28)	[63]
*Lus10024314*	*BZIP29*	bZIP transcription factor 29	Lu6_17695007	NUE_N+, R/S_N+	Root development	Down LN (−1.06)	[68]
*Lus10024908*	*NAC021*	NAC domain-containing protein 21/22	Lu9_19469655	R/S_N−, RV_Index	Root development	Up LN (1.20)	[69]
*Lus10031059*	*MIZ1*	Protein MIZU-KUSSEI 1	Lu9_6368499	NUE_N+	Root development	Down LN (−1.54), Down HN (−1.40)	[70]
*Lus10012461*	*CEPR2*	Receptor protein-tyrosine kinase CEPR2	Not in QTL	Unknown	Root development	Up LN (1.08)	[71]
*Lus10010238*	*MYB77*	Transcription factor MYB77	Not in QTL	Unknown	Root development	Down LN (−1.22)	[72]
*Lus10040274*	*LBD29*	LOB domain-containing protein 29	Not in QTL	Unknown	Root development	Up LN (1.80)	[73]
*Lus10025455*	*ACR4*	Serine/threonine-protein kinase-like protein ACR4	Not in QTL	Unknown	Root development	Up LN (1.63)	[74]
*Lus10040592*	*SUNN*	Leucine-rich repeat receptor-like kinase protein SUNN	Not in QTL	Unknown	Root development	Down LN (−1.32)	[75]
*Lus10017271*	*ABCB19*	ABC transporter B family member 19	Not in QTL	Unknown	Root development	Up LN (1.52)	[76]
*Lus10010012*	*ABCB4*	ABC transporter B family member 4	Not in QTL	Unknown	Root development	Down LN (−1.06)	[77]
*Lus10005574*	*CAT8*	Cationic amino acid transporter 8	Lu14_17132252	R/S_N+	Amino acid transport	Up LN (1.15)	[78]
*Lus10020181*	*GGCT2.1*	Gamma-glutamylcyclotransferase 2.1	Lu2_4220131	TRL_Index, RT_Index	Amino acid transport	Up LN (1.38)	[79]
*Lus10029533*	*BAT1*	Amino-acid permease BAT1 homolog	Not in QTL	Unknown	Amino acid transport	Up LN (1.12)	[80]
*Lus10012824*	*AVT1J*	Amino acid transporter AVT1J	Not in QTL	Unknown	Amino acid transport	Up LN (1.72)	[81]
*Lus10008910*	*GDU5*	Protein GLUTAMINE DUMPER 5	Not in QTL	Unknown	Amino acid transport	Down LN (−1.20), Down HN (−1.99)	[82]
*Lus10042740*	*AAP3*	Amino acid permease 3	Not in QTL	Unknown	Amino acid transport	Down LN (−1.49)	[83]
*Lus10011451*	*BAC2*	Mitochondrial arginine transporter BAC2	Not in QTL	Unknown	Amino acid transport	Down LN (−1.23)	[84]
*Lus10015513*	*GDU3*	Protein GLUTAMINE DUMPER 3	Not in QTL	Unknown	Amino acid transport	Down LN (−1.19)	[85]

LN: low NUE genotype; HN: high NUE genotype.

## Data Availability

The raw RNA-seq data were deposited in the NCBI under the bioproject PRJNA921700 (https://www.ncbi.nlm.nih.gov/bioproject/PRJNA921700 (accessed on 7 January 2023)).

## Supplementary Materials

The following supporting information can be downloaded at: https://www.mdpi.com/article/10.3390/ijms242417624/s1.
