# Supplementary material for: The Genetic Dissection of Nitrogen Use-Related Traits in Flax (Linum usitatissimum L.) at the Seedling Stage through the Integration of Multi-Locus GWAS, RNA-seq and Genomic Selection
Source: Int J Mol Sci. 2023 Dec 18;24(24):17624. doi: 10.3390/ijms242417624 (PMC10743809; doi:10.3390/ijms242417624)
Supplement: Supplementary file 1 [file ijms-24-17624-s001.zip › Supplementary material.pdf]

The Genetic Dissection of Nitrogen Use-Related Traits in Flax (*Linum usitatissimum* L.) at the Seedling Stage through the Integration of Multi-Locus GWAS, RNA-seq and Genomic Selection

Braulio J. Soto-Cerda [1,2,*], Giovanni Larama [3,4], Sylvie Cloutier [5], Bourlaye Fofana [6,*], Claudio Inostroza-Blancheteau [1,2] and Gabriela Aravena [1]

[1] Departamento de Ciencias Agropecuarias y Acuícolas, Universidad Católica de Temuco, Rudecindo Ortega 02950, Temuco 4781312, Chile
[2] Núcleo de Investigación en Producción Alimentaria, Facultad de Recursos Naturales, Universidad Católica de Temuco, Rudecindo Ortega 02950, Temuco 4781312, Chile
[3] Center of Plant, Soil Interaction and Natural Resources Biotechnology, Scientific and Technological Bioresource Nucleus, Universidad de La Frontera, Temuco 4811230, Chile
[4] Biocontrol Research Laboratory, Universidad de La Frontera, Temuco 4811230, Chile
[5] Ottawa Research and Development Centre, Agriculture and Agri-Food Canada, 960 Carling Avenue, Ottawa, ON K1A 0C6, Canada
[6] Charlottetown Research and Development Centre, Agriculture and Agri-Food Canada, 440 University Avenue, Charlottetown, PE C1A 4N6, Canada
* Correspondence: bsoto@uct.cl (B.J.S.-C.); bourlaye.fofana@agr.gc.ca (B.F.)

Supplementary material

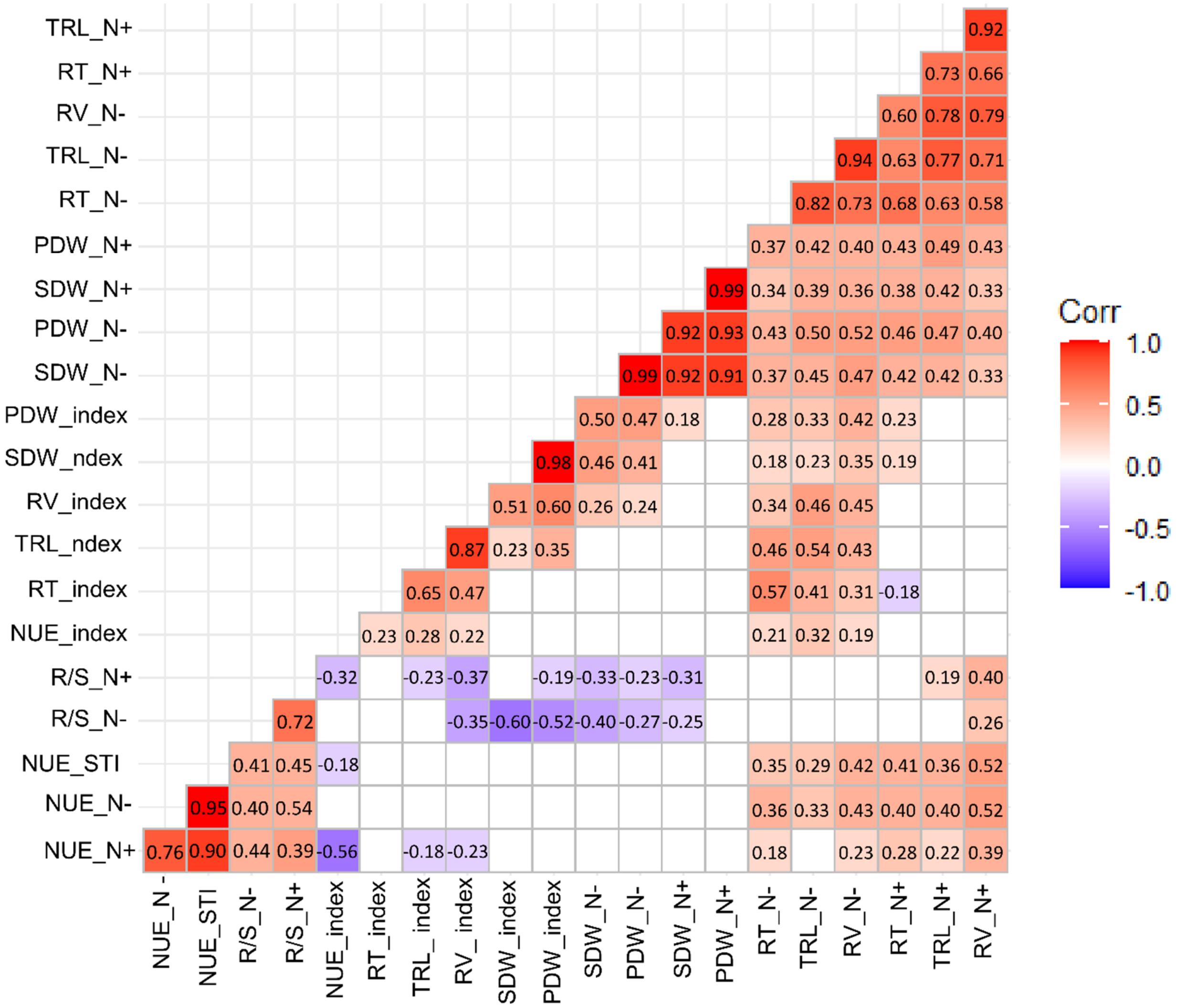

Figure S1. Pearson's correlations (P < 0.05) of 21 NUE-related traits under depleted and optimum N conditions. TRL_N-: Total root length N depleted; RV_N-: Root volume N depleted; RT_N-: Number of root tips N depleted; TRL_N+: Total root length N optimum; RV_N+: Root volume N optimum; RT_N+: Number of root tips N optimum; PDW_N-: Plant dry weight N depleted; SDW_N-: Shoot dry weight N depleted; PDW_N+: Plant dry weight N optimum; SDW_N+: Shoot dry weight N optimum; TRL_Index: Total root length stability index; RV_Index: Root volume stability index; RT_Index: Number of root tips stability index; PDW_Index: Plant dry weight stability index; SDW_Index: Shoot dry weight stability index; NUE_Index: Nitrogen use efficiency stability index; R/S_N-: Root to shoot ratio N depleted; R/S_N+: Root to shoot ratio N optimum; NUE_N-: Nitrogen use efficiency N depleted; NUE_N+: Nitrogen use efficiency N optimum; NUE_STI: Nitrogen use efficiency stress tolerance index.

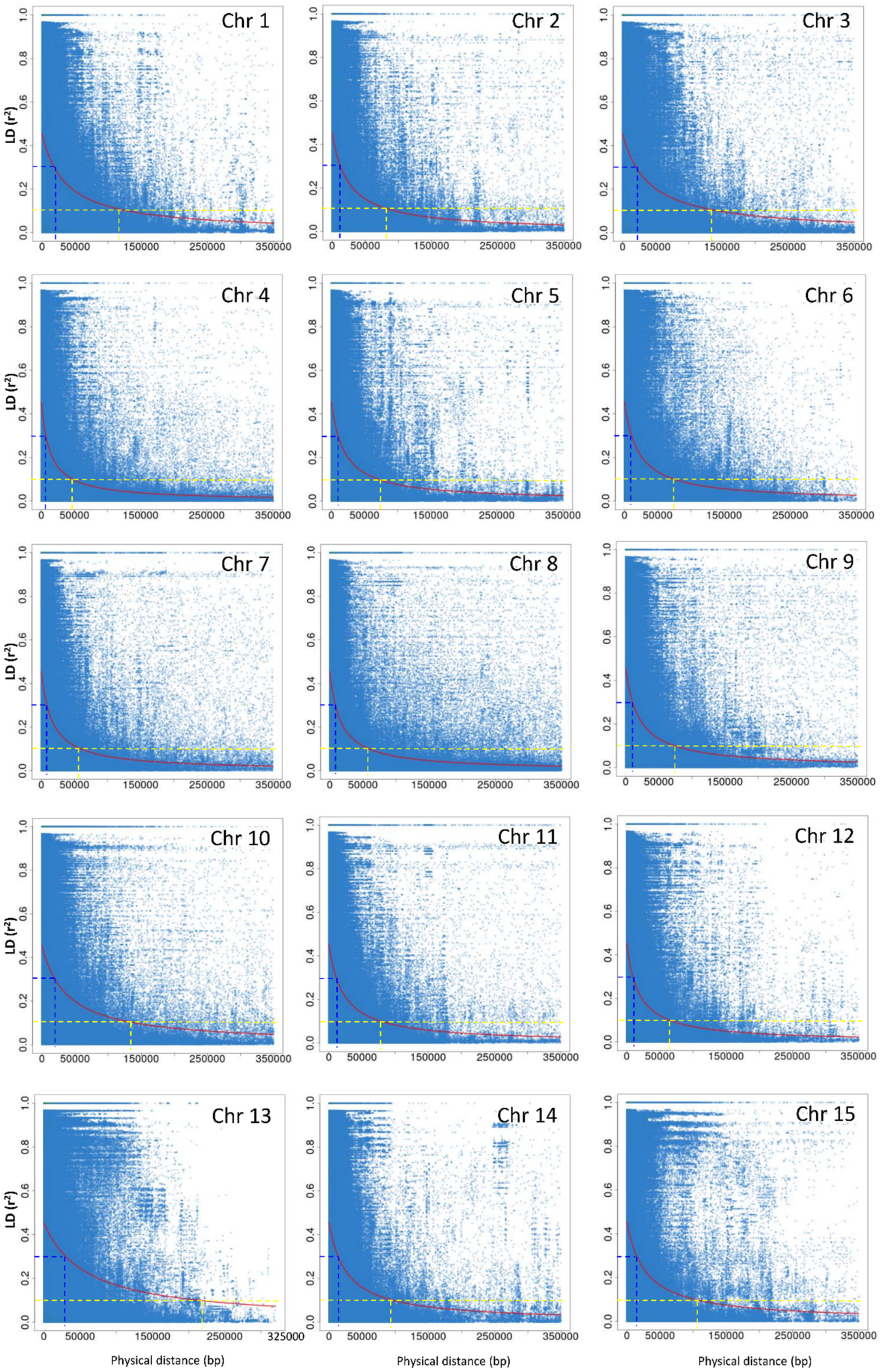

Figure S2. Linkage disequilibrium (LD) decay of r^2 values (red line), against physical distance (bp) across the 15 chromosomes of *Linum usitatissum*. Dashed blue and yellow lines indicate the cutoff value $r^2 = 0.3$ and $r^2 = 0.1$, respectively, used to group neighboring QTNs into QTL and the chromosome-specific LD decay.

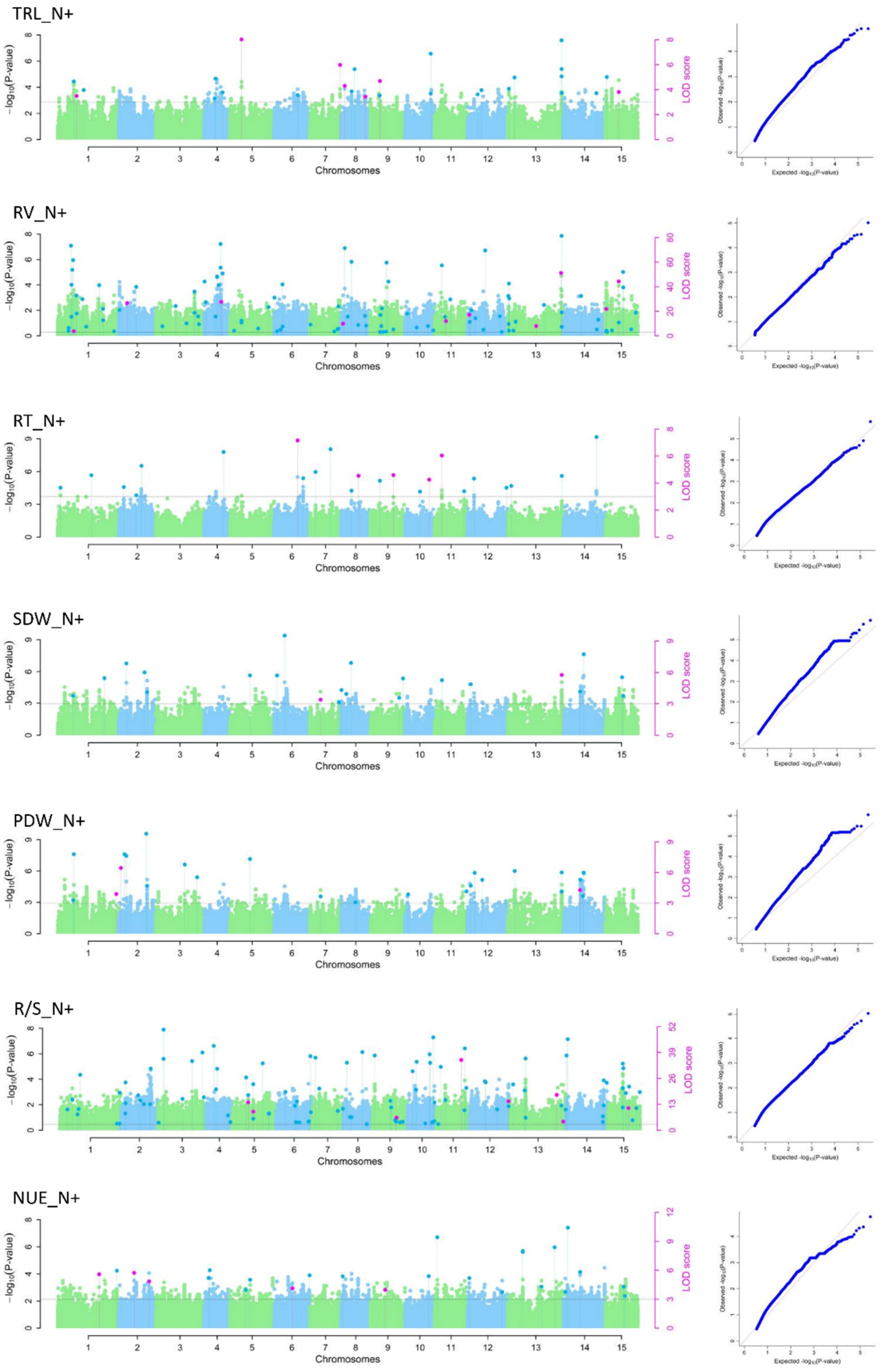
TRL_N+
RV_N+
RT_N+
SDW_N+
PDW_N+
R/S_N+
NUE_N+
$-\log_{10}$(P-value)
LOD score
Chromosomes
1 2 3 4 5 6 7 8 9 10 11 12 13 14 15
Expected $-\log_{10}$(P-value)
Observed $-\log_{10}$(P-value)

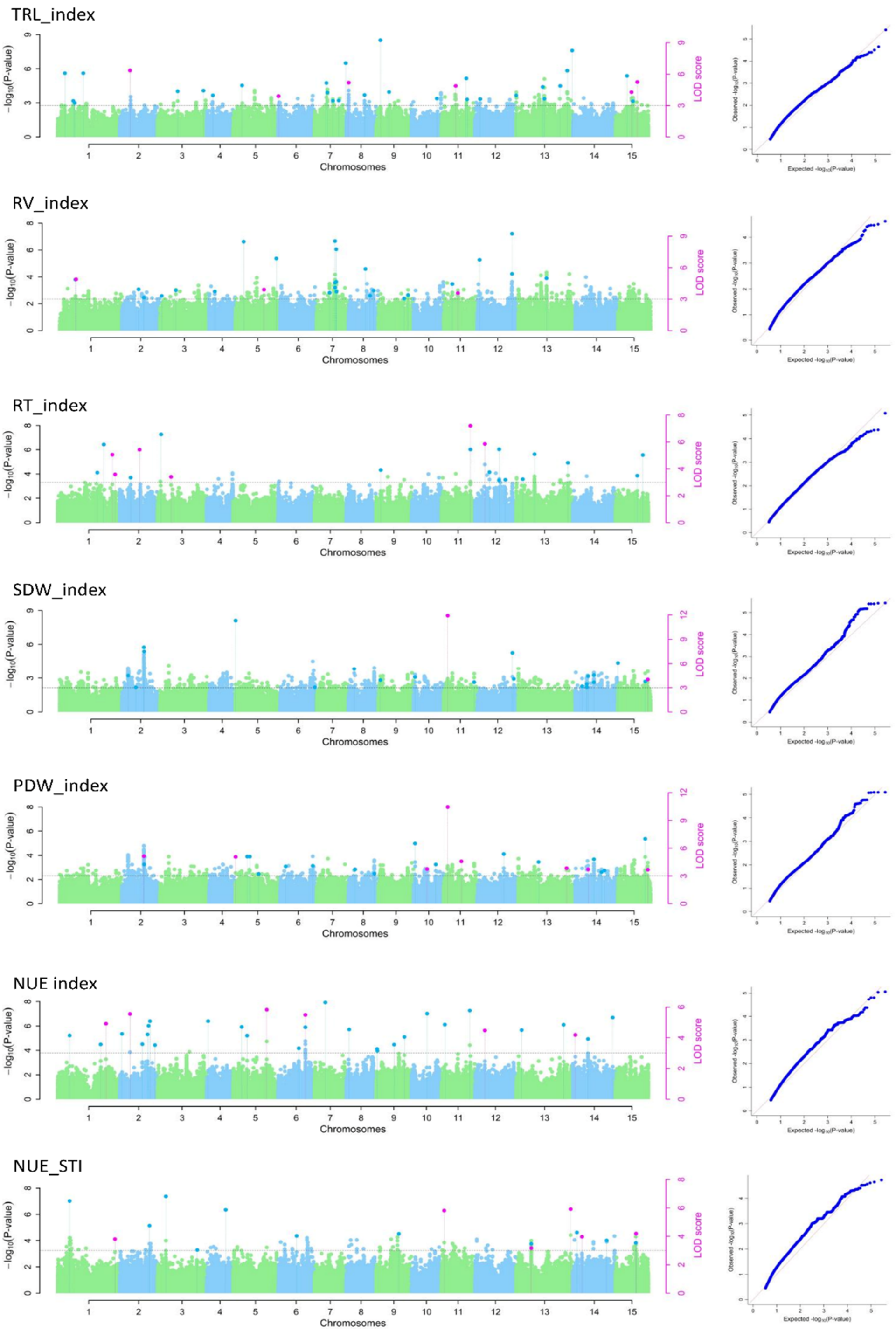

Figure S3. Manhattan (left panels) and quantile-quantile (Q-Q) plots (right panels) of multi-locus GWAS models of NUE-related traits. QTNs commonly identified by multiple approaches are indicated by the pink dots shown above dotted vertical lines; QTNs identified by a single model are represented by blue dots above vertical lines. The grey horizontal dotted line indicates the genome-wide significance threshold $-\log_{10}$ (P) equivalent to the LOD > 3.0 for multi-locus GWAS models. TRL_N+: Total root length N optimum, RV_N+: Root volume N optimum, RT_N+: Number of root tips N optimum, SDW_N+: Shoot dry weight N optimum, Plant dry weight N optimum, R/S_N+: Root to shoot ratio N optimum, NUE_N+: Nitrogen use efficiency N optimum. TRL_Index, RV_Index, RT_Index, SDW_Index, PDW_Index, and NUE_Index represent the stability indices for TRL, RV, RT, SDW, PDW and NUE measured under contrasting N conditions. NUE_STI: Nitrogen use efficiency stress tolerance index.

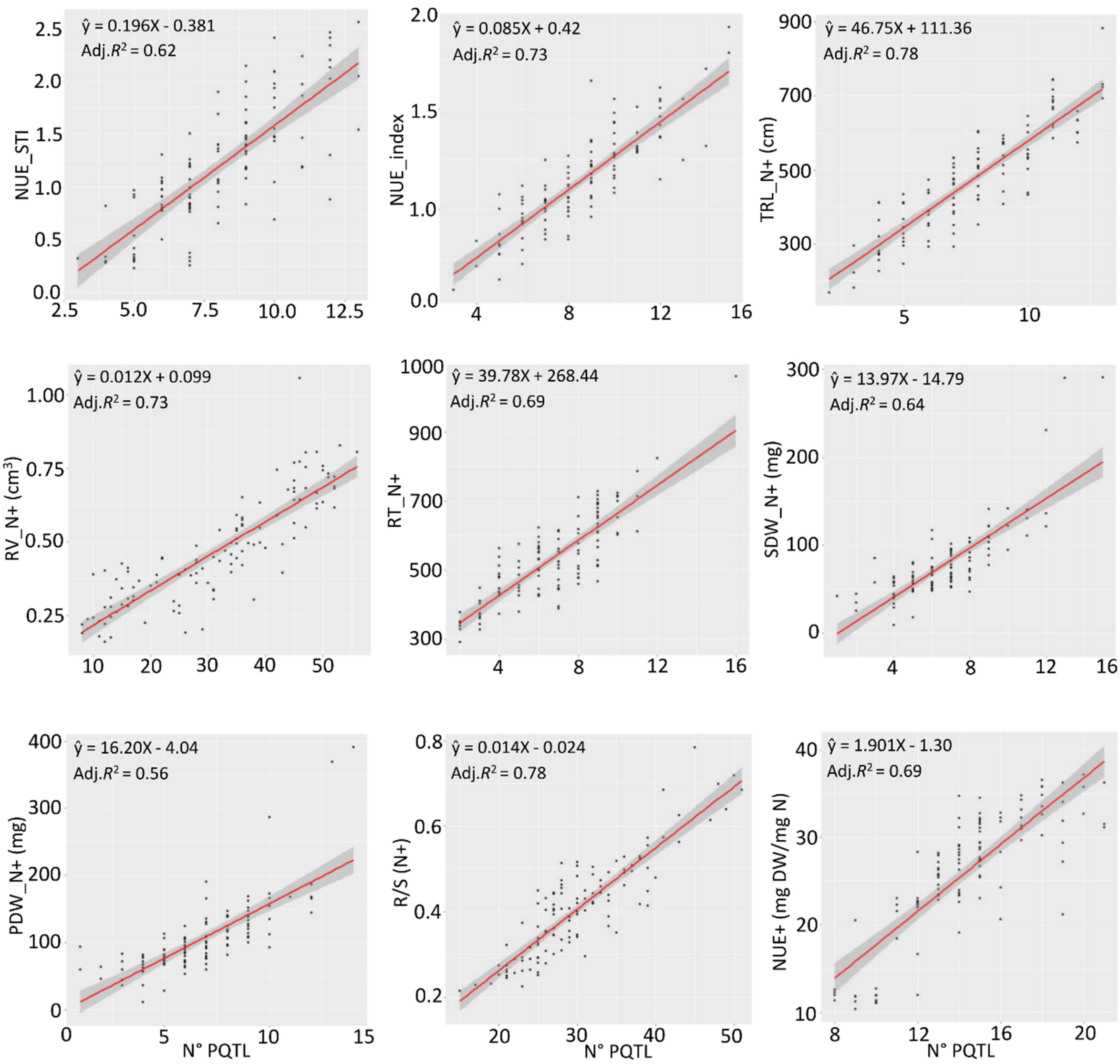

Figure S4. Simple regression analyses of QTL on trait for NUE-related traits and the phenotypic variation explained (adjR^2) for all detected QTL for each individual trait. NUE_STI: Nitrogen use efficiency stress tolerance index, NUE_Index: Nitrogen use efficiency index, TRL_N+: Total root length N optimum, RV_N+: Root volume N optimum, RT_N+: Number of root tips N optimum, SDW_N+: Shoot dry weight N optimum, Plant dry weight N optimum, R/S_N+: Root to shoot ratio N optimum, NUE_N+: Nitrogen use efficiency N optimum.

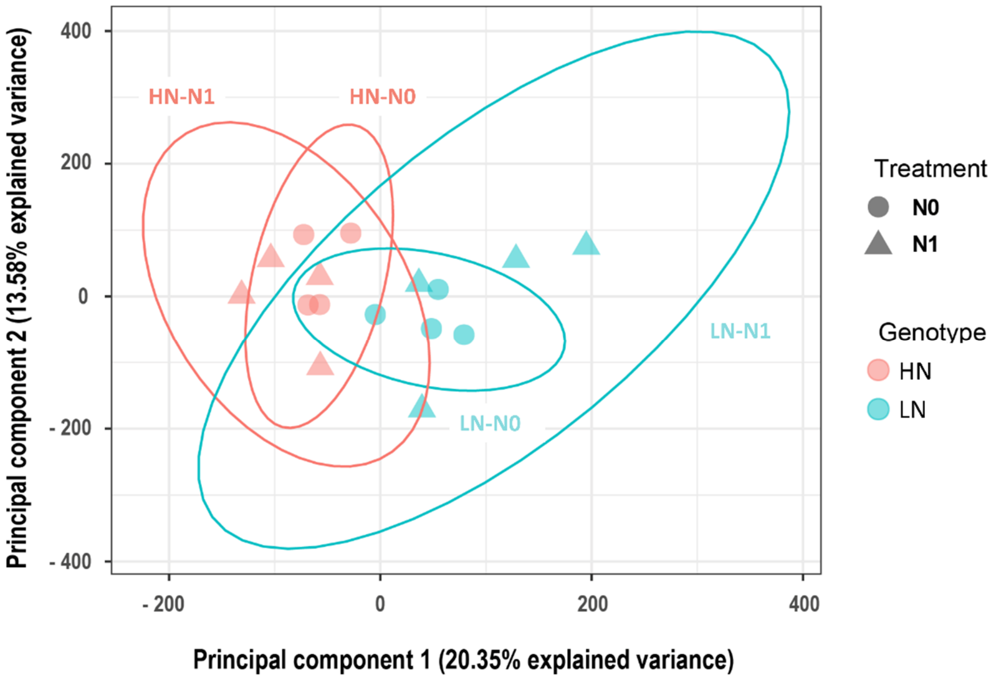

Figure S5. Principal component analysis (PCA) among the RNA-seq. samples generated under N contrasting conditions from four biological replicates per genotype. HN: High NUE genotype, LN: Low NUE genotype, N0: Depleted N treatment, N1: Optimum N treatment.

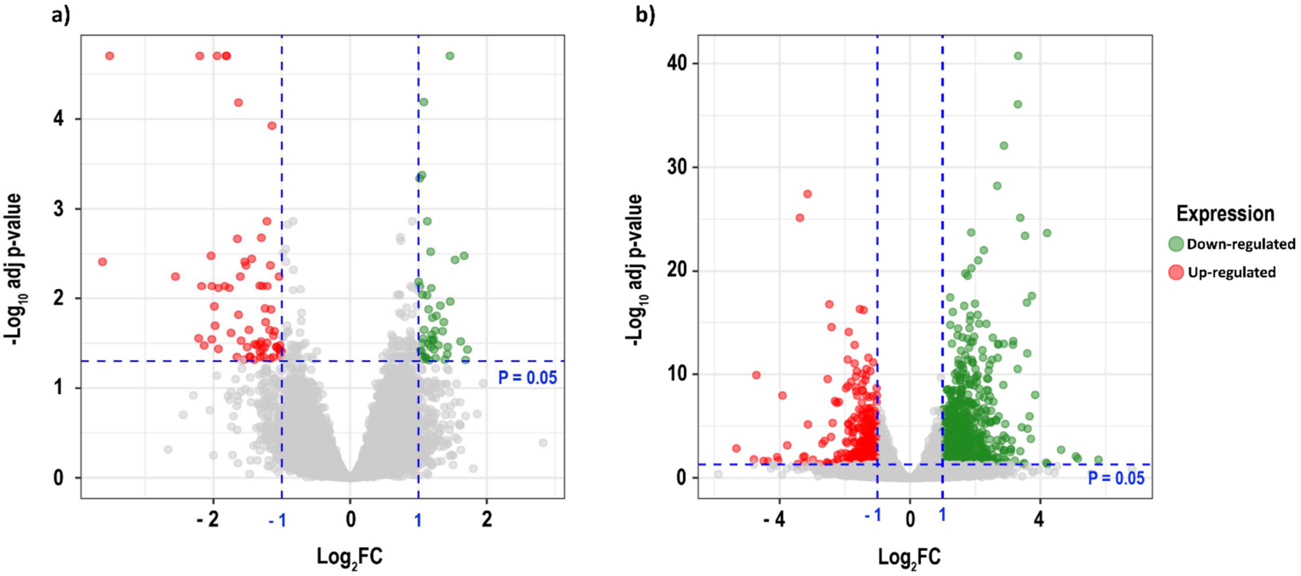

Figure S6. Volcano plots of up-regulated and down-regulated DEGs in roots under N contrasting conditions in flax. **a)** High NUE (HN) genotype. **b)** Low NUE (LN) genotype. Dotted vertical and horizontal blue lines indicate the $Log_2FC \geq 1$ and the FDR-adjusted P value < 0.05, respectively.

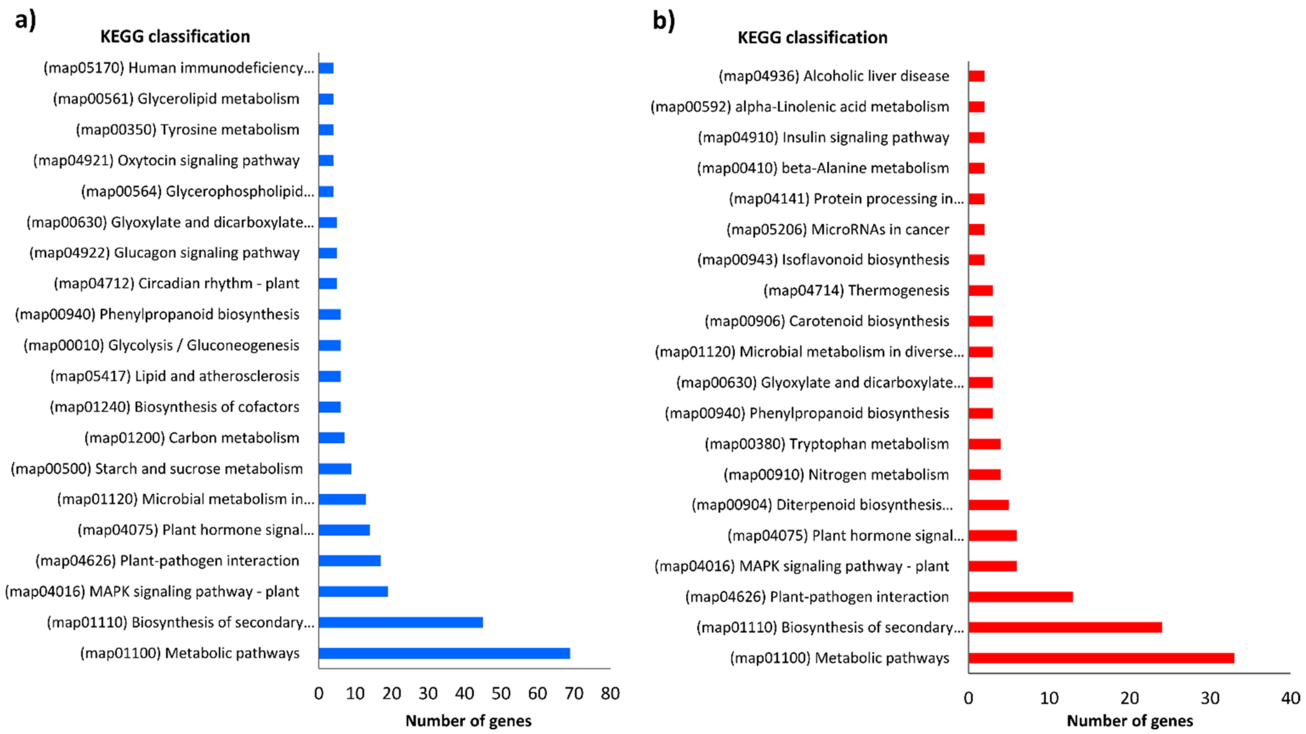

Figure S7. KEGG pathway annotation for the DEGs identified in root tissue for the LN genotype. (**a**) The twenty top KEGG enrichment pathways of up-regulated DEGs in LN genotype. (**b**) The twenty top KEGG pathways of down-regulated DEGs in LN genotype.

Table S1. Flax germplasm used for multi-locus GWAS analyses.

**Accession**	**Morphotype**	**Origin**
F_AFG_U_CN100952	Fiber	Afganistan
F_BLR_C_CN101038	Fiber	Belarus
F_CAN_C_G1186_94	Fiber	Canada
F_CHN_B_CN101416	Fiber	China
F_CZE_C_CN98704	Fiber	Czech Republic
F_EGY_C_CN98826	Fiber	Egypt
F_FRA_C_CN101392	Fiber	France
F_FRA_C_CN18986	Fiber	France
F_FRA_L_CN98710	Fiber	France
F_JPN_C_CN98072	Fiber	Japan
F_NLD_C_CN18987	Fiber	The Netherlands
F_NLD_C_CN18997	Fiber	The Netherlands
F_NLD_C_CN97424	Fiber	The Netherlands
F_NLD_U_CN101407	Fiber	The Netherlands
F_ROM_U_CN101405	Fiber	Romania
F_RUS_B_CN101039	Fiber	Russia
F_RUS_B_CN101114	Fiber	Russia
F_RUS_B_CN101116	Fiber	Russia
F_RUS_B_CN101127	Fiber	Russia
F_RUS_C_CN101099	Fiber	Russia
F_RUS_C_CN101154	Fiber	Russia
F_RUS_C_CN32542	Fiber	Russia
F_RUS_L_CN97503	Fiber	Russia
F_RUS_L_CN97531	Fiber	Russia
F_RUS_U_CN101348	Fiber	Russia
F_RUS_U_CN101394	Fiber	Russia
F_RUS_U_CN101396	Fiber	Russia
F_RUS_U_CN101402	Fiber	Russia
F_SWE_C_Atlas	Fiber	Sweden
F_TUR_U_CN101382	Fiber	Turkey
F_TUR_U_CN101386	Fiber	Turkey
F_UKR_U_CN101378	Fiber	Ukraine
F_UKR_U_CN101379	Fiber	Ukraine
F_UNK_C_CN33393	Fiber	Unknown
F_USA_B_CN98926	Fiber	United States of America
O_ARG_B_CN98037B	Oil	Argentina
O_ARG_C_CN97214	Oil	Argentina
O_ARG_C_CN97961	Oil	Argentina
O_ARG_C_CN97967	Oil	Argentina
O_ARG_C_CN98007	Oil	Argentina
O_ARG_C_CN98027	Oil	Argentina
O_ARG_C_CN98039	Oil	Argentina
O_AUS_C_CN98984	Oil	Australia
O_CAN_B_CN101496	Oil	Canada

O_CAN_B_CN101560	Oil	Canada
O_CAN_B_CN101596	Oil	Canada
O_CAN_B_CN101598	Oil	Canada
O_CAN_C_CDCGold	Oil	Canada
O_CAN_C_CN101413	Oil	Canada
O_CAN_C_CN18981	Oil	Canada
O_CAN_C_CN19003	Oil	Canada
O_CAN_C_CN19004	Oil	Canada
O_CAN_C_CN19159	Oil	Canada
O_CAN_C_CN33388	Oil	Canada
O_CAN_C_CN33389	Oil	Canada
O_CAN_C_CN52732	Oil	Canada
O_CAN_C_CN97633	Oil	Canada
O_CAN_C_Macbeth	Oil	Canada
O_CAN_C_Shape	Oil	Canada
O_DEU_B_CN97430	Oil	Germany
O_DEU_C_CN100881	Oil	Germany
O_DEU_C_CN97886	Oil	Germany
O_FRA_B_CN100863	Oil	France
O_GBR_C_CN101265	Oil	United Kingdom
O_GEO_U_CN101366	Oil	Georgia
O_HUN_C_CN100883	Oil	Hungary
O_HUN_C_CN97287	Oil	Hungary
O_HUN_C_CN97300	Oil	Hungary
O_HUN_C_CN98263	Oil	Hungary
O_HUN_C_CN98263B	Oil	Hungary
O_HUN_C_CN98275	Oil	Hungary
O_HUN_C_CN98854	Oil	Hungary
O_IND_C_CN101208	Oil	India
O_IND_C_CN98057	Oil	India
O_IND_C_CN98135	Oil	India
O_IND_C_CN98250	Oil	India
O_IND_C_CN98982	Oil	India
O_IRL_C_CN98192	Oil	Ireland
O_JPN_C_CN97470	Oil	Japan
O_LTU_B_CN101240	Oil	Lithuania
O_LTU_C_CN101237	Oil	Lithuania
O_MAR_B_CN101026	Oil	Morocco
O_NLD_C_CN18993	Oil	The Netherlands
O_NLD_C_CN98056	Oil	The Netherlands
O_NLD_C_CN98056B	Oil	The Netherlands
O_PAK_C_CN97064	Oil	Pakistan
O_PAK_C_CN97096	Oil	Pakistan
O_PAK_C_CN97103	Oil	Pakistan
O_PAK_C_CN98237	Oil	Pakistan

O_PAK_C_CN98239	Oil	Pakistan
O_ROM_C_CN100674	Oil	Romania
O_RUS_B_CN101241	Oil	Russia
O_RUS_B_CN101301	Oil	Russia
O_RUS_C_CN96846	Oil	Russia
O_RUS_C_CN97475	Oil	Russia
O_RUS_C_CN97489	Oil	Russia
O_RUS_C_CN97529	Oil	Russia
O_TUR_U_CN101331	Oil	Turkey
O_UKR_C_CN30860	Oil	Ukraine
O_UNK_C_CN100547	Oil	Unknown
O_UNK_C_CN30861	Oil	Unknown
O_URY_C_CN98100	Oil	Uruguay
O_USA_B_CN98566	Oil	United States of America
O_USA_C_CN18994	Oil	United States of America
O_USA_C_CN33399	Oil	United States of America
O_USA_C_CN33400	Oil	United States of America
O_USA_C_CN33992	Oil	United States of America
O_USA_C_CN97377	Oil	United States of America
O_USA_C_CN97396	Oil	United States of America
O_USA_C_CN97463	Oil	United States of America
O_USA_C_CN97642	Oil	United States of America
O_USA_C_CN97718	Oil	United States of America
O_USA_C_CN98542	Oil	United States of America
U_ETH_L_CN100895B	Unknown	Ethiopia
U_MAR_C_CN98193	Unknown	Morocco
U_NZL_B_CN100797B	Unknown	New Zeland
U_ROM_C_CN100678	Unknown	Romania
U_RUS_L_CN97483	Unknown	Russia
U_TUR_U_CN100828	Unknown	Turkey
U_USA_B_CN97406	Unknown	United States of America
U_USA_B_CN98644	Unknown	United States of America
U_USA_C_CN97397	Unknown	United States of America

C = Cultivar, B = Breeding line, L= Landrace, U = Unknown improvement status. CN = Canadian number at Plant Gene Resources of Canada (PGRC)

Table S2. Restricted maximum likelihood analysis (REML) of root and shoot traits in the flax panel

Trait	Treatment Wald statistic	Genotype Wald statistic	Treatment x Genotype Wald statistic
Total root length	< 0.001	< 0.001	0.606
Total root volume	< 0.001	< 0.001	0.581
Number of root tips	< 0.001	< 0.001	0.106
Plant dry weight	< 0.001	< 0.001	0.453
Shoot dry weight	< 0.001	< 0.001	< 0.001
Root dry weight	0.526	< 0.001	0.340
Root / shoot	< 0.001	< 0.001	0.926

Total root length (cm), Total root volume (cm^3), Number of root tips, Plant dry weight (mg), Shoot dry weight (mg), Root dry weight (mg), Root / shoot (%)

Table S3. List of 359 NUE-related QTL identified by multi-locus GWAS methods and the differentially expressed candidate genes located within ± 100 kb of the tag QTNs

Tag QTN	Chr/Pos	Trait	Method*	$-Log_{10}$ (P) range	MAF	R^2 (%)	Genotype	DEG
Lu1_183477	1/183477	R/S_N-	1, 4	7.96 – 8.05	0.150	4.99	AA	-
Lu1_502406	1/502406	R/S_N-	3	4.71	0.305	0.68	AA	-
Lu1_1839328	1/1839328	TRL_Index	1	6.92	0.169	5.27	GG	-
Lu1_3301944	1/3301944	TRL_N-	1	3.92	0.256	6.01	AA	*Lus10042369*
Lu1_3381304	1/3381304	RV_N+	2	7.30	0.480	0.44	TT	-
Lu1_3815901	1/3815901	RV_N-	6	7.18	0.337	0.83	AA	-
Lu1_3872648	1/3872648	NUE_STI	4	7.33	0.171	0.49	GG	-
Lu1_4123820	1/4123820	R/S_N+	2	16.41	0.094	0.49	AA	*Lus10008643*
Lu1_4360783	1/4360783	RV_N+, RV_N-	2, 6	4.79 – 74.74	0.207	2.39	CC	-
Lu1_4538185	1/4538185	RV_N+	2	16.62 – 42.86	0.256	1.01	AA	-
Lu1_4965387	1/4965387	RV_N+	2	55.03	0.484	0.04	CC	*Lus10002807*, *Lus10002834*, *Lus10012064*
Lu1_5242415	1/5242415	RV_N+, RT_N-	1, 2	3.78 – 63.01	0.153	5.81	GG	*Lus10012002*, *Lus10012035*
Lu1_5660224	1/5660224	RV_Index	1, 2, 6	4.34 – 5.74	0.126	4.19	CC	*Lus10012546*
Lu1_5688683	1/5688683	TRL_N+, PDW_N+	1, 2	5.43 – 8.51	0.142	9.55	GG	-
Lu1_6326887	1/6326887	R/S_N+	2	8.84	0.073	0.23	CC	*Lus10034310*, *Lus10034298*
Lu1_6744808	1/6744808	R/S_N+, TRL_N+, RV_N+	1, 2	4.35 – 33.92	0.223	8.10	AA	-
Lu1_9592260	1/9592260	RV_N+	2	31.03	0.272	0.83	CC	*Lus10004226*
Lu1_9831112	1/9831112	TRL_Index	1	6.92	0.174	8.68	AA	*Lus10022748*, *Lus10022736*
Lu1_10175480	1/10175480	SDW_N-, PDW_N-, SDW_N+, TRL_N+	1, 2, 3, 6	4.41–10.20	0.431	5.24	TT	*Lus10022675*
Lu1_12954522	1/12954522	RT_N+	2	4.84	0.386	4.62	AA	*Lus10005204*
Lu1_16899889	1/16899889	RT_Index	3	4.45	0.382	9.46	CC	-
Lu1_17639403	1/17639403	RV_N-	1, 2, 3	4.38 – 8.32	0.260	6.30	AA	-
Lu1_17958777	1/17958777	RV_N+, NUE+	1, 2, 6	6.11 – 42.47	0.333	6.32	AA	-
Lu1_18006936	1/18006936	TRL_N-, RV_N-	1, 2, 4, 6	3.92 – 6.11	0.289	4.14	CC	-
Lu1_18475694	1/18475694	NUE_Index	4	4.29	0.138	1.81	GG	-
Lu1_19247653	1/19247653	SDW_N+	6	3.75	0.289	0.99	AA	-
Lu1_19536167	1/19536167	R/S_N-	6	5.29	0.447	0.37	CC	-
Lu1_19572730	1/19572730	RV_N+	2	22.98	0.313	1.87	GG	-
Lu1_19697968	1/19697968	RT_Index	4	6.64	0.094	5.04	GG	-
Lu1_20419533	1/20419533	RT_N-	2	5.42	0.252	3.26	GG	-
Lu1_20935186	1/20935186	NUE_Index	2, 4	5.14 – 6.27	0.098	2.86	TT	-
Lu1_22850090	1/22850090	PDW_N+	6	4.92	0.098	0.84	GG	-
Lu1_25965118	1/25965118	RT_Index	2, 6	4.72 – 5.28	0.163	3.91	AA	*Lus10006547*, *Lus10006552*, *Lus10006563*
Lu1_27500392	1/27500392	NUE_STI, RT_Index	1, 2, 4, 6	3.96 – 5.60	0.187	6.00	AA	-

Lu1_27734323	1/27734323	R/S_N-, R/S_N+	6	3.98 – 4.73	0.264	2.03	CC	-
Lu1_28257219	1/28257219	PDW_N+	3,6	4.30 – 5.37	0.215	3.96	TT	-
Lu1_28369667	1/28369667	NUE+	4	6.79	0.407	6.74	CC	*Lus10018926*
Lu1_28527080	1/28527080	R/S_N+	4	4.01	0.207	1.25	TT	*Lus10000771*
Lu1_28827671	1/28827671	RT_N+	6	5.43	0.122	1.93	AA	*Lus10006075*
Lu1_28921963	1/28921963	RT_N-, NUE-	1, 2, 6	4.46 – 8.19	0.094	12.80	GG	-
Lu2_783617	2/783617	TRL_N-, NUE-	1, 4	3.83 – 6.56	0.087	11.78	AA	*Lus10019256*
Lu2_1311870	2/1311870	R/S_N-	2, 4	4.26 – 12.13	0.191	5.28	AA	-
Lu2_2232215	2/2232215	R/S_N-, R/S_N+	2, 3	7.49 – 9.32	0.313	1.41	AA	*Lus10016358*, *Lus10016353*
Lu2_2368527	2/2368527	R/S_N+	2	14.58 – 25.10	0.114	0.85	GG	*Lus10016331*
Lu2_2727588	2/2727588	R/S_N-	3	6.93	0.423	3.18	CC	*Lus10002324*
Lu2_3069270	2/3069270	R/S_N-	4	6.44	0.211	11.79	GG	*Lus10014352*, *Lus10014340*
Lu2_3641485	2/3641485	RV_N+	2	45.50	0.215	0.28	TT	*Lus10003439*
Lu2_4053688	2/4053688	TRL_Index, NUE_Index	1, 2, 4	4.15 – 8.55	0.065	4.88	CC	-
Lu2_4220131	2/4220131	TRL_Index, RT_Index	4, 6	4.06 – 7.48	0.203	7.01	AA	*Lus10020193*, *Lus10020174*, *Lus10020181*
Lu2_4461069	2/4461069	PDW_N-, RV_N-, TRL_N-	1, 2, 3, 6	5.71 – 12.59	0.488	9.09	CC	-
Lu2_5142092	2/5142092	SDW_Index	1	3.73	0.149	9.5	GG	-
Lu2_5901183	2/5901183	RV_N-, RT_N-	3, 4	4.43 – 6.03	0.309	4.40	GG	-
Lu2_5928955	2/5928955	NUE+	3, 4	5.10 – 8.00	0.459	8.59	AA	-
Lu2_6861078	2/6861078	RV_N+	2	41.15	0.167	2.23	TT	-
Lu2_6903912	2/6903912	RT_N+	2	5.16	0.126	4.56	TT	-
Lu2_7470418	2/7470418	SDW_N+	6	5.84	0.089	1.93	CC	-
Lu2_7575890	2/7575890	PDW_N-	6	4.64	0.187	1.62	GG	-
Lu2_9463028	2/9463028	RT_Index	1, 2, 4, 6	3.72 – 9.96	0.065	7.53	GG	-
Lu2_19111900	2/19111900	SDW_Index, PDW_Index, RV_Index	2, 4, 6	3.85 – 8.91	0.163	7.50	TT	*Lus10021827*
Lu2_19272779	2/19272779	NUE_Index	2	4.31	0.085	0.02	GG	-
Lu2_19401332	2/19401332	SDW_N-, PDW_N-, R/S_N-	2, 4, 6	4.31 – 10.52	0.102	4.78	GG	-
Lu2_19451785	2/19451785	R/S_N+	2	14.09	0.094	0.86	CC	-
Lu2_21668160	2/21668160	PDW_N+, NUE_Index	2, 4	4.98 – 9.03	0.130	7.51	TT	*Lus10035126*
Lu2_21694722	2/21694722	R/S_N-, R/S_N+	2, 6	5.14 – 5.63	0.155	2.53	CC	-

Lu2_21834746	2/21834746	SDW_N+, PDW_N+, NUE_Index	4, 6	4.85 – 5.56	0.138	12.43	TT	*Lus10035175*
Lu2_21943322	2/21943322	R/S_N+, NUE_STI	2	5.54 – 14.08	0.094	3.86	AA	-
Lu2_22168558	2/22168558	NUE_Index, R/S_N+	2	5.88 – 32.08	0.081	0.93	GG	-
Lu2_22306950	2/22306950	NUE+	1, 6	4.46 – 6.80	0.225	5.95	GG	-
Lu2_23979210	2/23979210	NUE_Index	6	4.24	0.106	0.02	GG	*Lus10030589*
Lu3_964017	3/964017	RV_Index	1	4.02	0.116	14.54	GG	*Lus10021939*
Lu3_1295318	3/1295318	RT_Index	2	7.41	0.065	8.00	CC	*Lus10037389*
Lu3_1536734	3/1536734	RV_N-	6	4.22	0.240	0.88	GG	-
Lu3_1823319	3/1823319	R/S_N+	2, 6	5.56 – 51.71	0.297	3.29	TT	-
Lu3_2936603	3/2936603	RT_N+	6	6.09	0.089	3.20	AA	-
Lu3_3532734	3/3532734	NUE_STI	2	7.67	0.130	7.33	CC	*Lus10019190*, *Lus10000642*
Lu3_5585030	3/5585030	RT_Index	2, 4	4.05 – 4.19	0.431	2.81	AA	-
Lu3_6931619	3/6931619	RV_Index	4	4.59	0.472	5.77	AA	*Lus10040821*
Lu3_8280597	3/8280597	TRL_Index, RV_N+	2	5.13 – 25.40	0.155	5.41	GG	*Lus10001885*
Lu3_14389194	3/14389194	R/S_N+	2	16.74	0.069	2.20	GG	-
Lu3_16915609	3/16915609	SDW_N+, PDW_N+	2	3.79 – 8.43	0.106	5.25	GG	-
Lu3_17130944	3/17130944	SDW_N+	6	4.42	0.248	1.41	CC	-
Lu3_19038225	3/19038225	R/S_N+	2	35.85	0.106	0.53	GG	-
Lu3_19482873	3/19482873	RT_N-	6	3.81	0.309	1.80	CC	-
Lu3_20904140	3/20904140	RV_N+	2	19.81 – 37.25	0.195	0.91	CC	-
Lu3_21020734	3/21020734	NUE-, NUE_STI	1, 2	3.74 – 4.88	0.168	7.78	CC	-
Lu3_22332646	3/22332646	SDW_N-, PDW_N-	6	3.79 – 6.62	0.150	1.74	TT	-
Lu3_25055317	3/25055317	R/S_N+	2	17.59	0.094	1.81	TT	*Lus10037644*
Lu4_158148	4/158148	RV_N+	2	45.48	0.366	0.20	GG	-
Lu4_387643	4/387643	NUE_Index	2	5.88	0.220	2.58	AA	-
Lu4_434920	4/434920	RV_N+	2	28.62	0.106	0.57	CC	-
Lu4_893602	4/893602	RT_N-	6	6.34	0.333	6.18	AA	-
Lu4_1182364	4/1182364	NUE+	6	6.00	0.126	2.10	AA	-
Lu4_2006452	4/2006452	NUE+, RV_Index	4, 6	4.47 – 6.84	0.488	3.83	TT	*Lus10009847*
Lu4_2103588	4/2103588	TRL_Index	2, 6	4.22 – 4.72	0.447	3.89	CC	-
Lu4_8268087	4/8268087	SDW_N-	1	4.00	0.157	3.93	GG	*Lus10039504*
Lu4_8780339	4/8780339	SDW_N-	1	4.89	0.062	6.98	AA	*Lus10029002*
Lu4_11529805	4/11529805	TRL_N+, RV_N+	2, 4	5.67 – 16.73	0.240	2.08	AA	-
Lu4_12589151	4/12589151	TRL_N-	1	3.81	0.141	11.19	CC	*Lus10036941*
Lu4_12792147	4/12792147	RV_N+	2	49.09 – 49.63	0.126	1.61	TT	*Lus10036965*
Lu4_13634659	4/13634659	RV_N+, SDW_N-	2, 6	3.91 – 42.51	0.252	3.29	TT	*Lus10034407*

Lu4_14298191	4/14298191	R/S_N-	3	6.08	0.228	1.31	CC	*Lus10041444*, *Lus10041466*, *Lus10041461*, *Lus10041473*, *Lus10041460*
Lu4_14523601	4/14523601	RV_N+	2	56.83 – 76.15	0.114	0.69	AA	*Lus10041489*, *Lus10041487*
Lu4_14642224	4/14642224	RV_N+	2	52.45	0.073	0.11	CC	*Lus10041543*, *Lus10041526*
Lu4_14879748	4/14879748	R/S_N-	4	3.79	0.175	5.99	TT	-
Lu4_15074000	4/15074000	RV_N+	2	52.10	0.281	0.11	CC	*Lus10041628*, *Lus10041614*, *Lus10041616*, *Lus10041622*
Lu4_15303541	4/15303541	NUE_STI	2	6.69	0.155	4.94	CC	*Lus10041664*
Lu4_16158145	4/16158145	RV_N-	6	6.00	0.195	1.23	AA	*Lus10041830*, *Lus10041843*
Lu4_17131980	4/17131980	RT_N+, SDW_N-, PDW_N-	1, 2, 6	6.52 – 8.01	0.293	12.24	GG	-
Lu4_17401223	4/17401223	RV_N-	6	7.74	0.236	0.39	CC	-
Lu4_17463420	4/17463420	RV_N-	6	12.10	0.240	0.66	GG	*Lus10020728*
Lu4_17943761	4/17943761	R/S_N-	6	4.90	0.073	1.92	CC	*Lus10020658*
Lu4_18720426	4/18720426	RV_N-	6	4.63	0.366	0.15	TT	*Lus10027185*, *Lus10027172*, *Lus10030042*, *Lus10030044*
Lu4_19049038	4/19049038	R/S_N+	2	4.82	0.106	0.80	AA	Lus10039710, Lus10039725, Lus10039732
Lu5_88517	5/88517	SDW_Index, PDW_Index	1, 2, 4, 6	3.95 – 12.27	0.431	9.38	GG	-
Lu5_2098651	5/2098651	RV_Index	1	9.38	0.265	14.40	AA	-
Lu5_2166168	5/2166168	NUE_Index	2	5.49	0.301	0.10	TT	-
Lu5_2260383	5/2260383	TRL_Index	1	5.71	0.310	7.99	TT	-
Lu5_2951268	5/2951268	TRL_N+, RV_N+, RV_N-, PDW_Index	1, 2, 4, 6	5.41 – 13.42	0.199	8.48	CC	*Lus10032303*, *Lus10032316*
Lu5_3595413	5/3595413	R/S_N-, NUE_Index, R/S_N+	2, 3, 6	4.90 – 29.83	0.472	0.53	CC	*Lus10032421*, *Lus10033226*, *Lus10033223*
Lu5_4220146	5/4220146	PDW_N+	6	4.60	0.065	0.83	AA	-
Lu5_4528185	5/4528185	NUE+	4	4.72	0.382	3.58	CC	*Lus10034779*
Lu5_4628966	5/4628966	R/S_N+	2	24.23	0.476	0.41	CC	-
Lu5_6218765	5/6218765	NUE+	1	5.81	0.248	12.34	AA	-
Lu5_8169998	5/8169998	R/S_N+	2	6.60 – 24.22	0.061	0.42	GG	-
Lu5_9381335	5/9381335	PDW_Index	2	3.94	0.069	1.82	AA	-
Lu5_11945444	5/11945444	RV_N+, RV_Index	2, 3	3.96 – 6.79	0.106	4.97	AA	*Lus10035293*, *Lus10035284*, *Lus10035289*
Lu5_12191977	5/12191977	SDW_N-, PDW_N-	1	4.23 – 6.36	0.331	8.86	CC	-
Lu5_14527440	5/14527440	NUE_Index	2, 6	5.14 – 8.15	0.089	5.32	GG	*Lus10028453*, *Lus10028463*
Lu5_16368000	5/16368000	RT_N-, RV_N+	2, 6	4.21 – 24.53	0.211	5.59	AA	-
Lu5_16695813	5/16695813	RV_Index	2	7.73	0.305	7.35	CC	*Lus10039666*
Lu5_17289922	5/17289922	TRL_N+	6	4.43	0.439	2.21	CC	*Lus10018533*, *Lus10018530*, *Lus10018532*

Lu6_252613	6/252613	RV_N+	2	32.30	0.325	0.21	CC	-
Lu6_269306	6/269306	RV_N-, TRL_N-, TRL_Index	2, 4, 6	4.18 – 7.28	0.228	3.89	GG	*Lus10010943, Lus10010940, Lus10010930, Lus10010927*
Lu6_1634298	6/1634298	TRL_N-	4	5.34	0.163	3.72	TT	*Lus10017723*
Lu6_3262361	6/3262361	RV_N-	6	5.25	0.089	0.89	AA	*Lus10019494, Lus10019463, Lus10019490*
Lu6_4289550	6/4289550	SDW_N-, PDW_N-	1	4.14 – 5.96	0.438	11.88	GG	-
Lu6_4400099	6/4400099	RV_N+	2	43.14	0.260	0.82	CC	-
Lu6_4642295	6/4642295	RV_N+	2	8.52	0.142	1.06	GG	-
Lu6_5225959	6/5225959	R/S_N+	2	20.28	0.130	0.94	TT	-
Lu6_5700373	6/5700373	SDW_N-, PDW_N-, SDW_N+, PDW_N+	2, 6	4.91 – 10.89	0.081	5.55	AA	-
Lu6_5719475	6/5719475	SDW_N-	4	4.40	0.073	16.92	AA	-
Lu6_9232719	6/9232719	RV_N-	6	10.88	0.447	0.79	GG	-
Lu6_12083087	6/12083087	R/S_N-, NUE_STI, NUE+	2, 4, 6	4.33 – 5.12	0.309	4.54	AA	-
Lu6_12435808	6/12435808	R/S_N+	2	13.23	0.146	0.69	AA	*Lus10016062*
Lu6_13245642	6/13245642	R/S_N+, NUE_Index	2	4.03 – 4.87	0.106	1.75	TT	*Lus10013841, Lus10013827*
Lu6_13654293	6/13654293	R/S_N+	2	15.37	0.073	0.68	GG	-
Lu6_15405962	6/15405962	NUE_Index	2, 4	4.06 – 8.51	0.155	3.03	AA	*Lus10021019, Lus10021010*
Lu6_16074953	6/16074953	RT_N+	4, 6	5.13 – 8.56	0.220	5.08	GG	-
Lu6_16240613	6/16240613	R/S_N-	2	5.55	0.248	1.73	CC	-
Lu6_16828246	6/16828246	PDW_Index, R/S_N+	1, 2	4.85 – 5.22	0.372	13.01	AA	*Lus10025252, Lus10025265, Lus10025269, Lus10024438*
Lu6_17122009	6/17122009	TRL_Index	6	4.95	0.187	5.35	TT	-
Lu6_17318908	6/17318908	SDW_Index, R/S_N+	1, 2	3.75 – 38.37	0.079	11.60	CC	*Lus10024367*
Lu6_17695007	6/17695007	R/S_N+, NUE+	2, 6	4.00 – 6.29	0.394	6.81	GG	*Lus10024314, Lus10024306*
Lu7_406563	7/406563	RV_N+	2	10.10	0.443	0.13	GG	*Lus10017362, Lus10017372*
Lu7_1606466	7/1606466	R/S_N+	2	37.60	0.215	0.49	GG	*Lus10028791*
Lu7_2612672	7/2612672	RT_N+	4	5.61	0.281	3.85	AA	-
Lu7_3162503	7/3162503	R/S_N+	2, 6	7.08 – 21.96	0.114	2.78	GG	*Lus10023525*
Lu7_4527136	7/4527136	TRL_Index	3	5.96	0.195	10.66	GG	*Lus10040179*
Lu7_4646757	7/4646757	R/S_N-	3	5.04	0.439	0.93	CC	*Lus10040134*
Lu7_4861539	7/4861539	TRL_Index	2	5.00	0.407	5.93	GG	-
Lu7_7173159	7/7173159	TRL_Index, RV_Index	2, 4, 6	4.19 – 9.44	0.102	8.76	CC	*Lus10024457*
Lu7_9137683	7/9137683	RV_Index	2, 6	4.25 – 8.63	0.163	6.88	CC	-
Lu7_9194656	7/9194656	RV_Index	4	5.43	0.191	7.01	GG	-
Lu7_15677858	7/15677858	TRL_Index	2	4.21	0.236	1.72	GG	-

Lu7_17139409	7/17139409	RV_N+	2	25.10	0.089	0.41	TT	-
Lu7_17880184	7/17880184	R/S_N-, TRL_N+	1, 2, 4, 6	4.64 – 7.85	0.471	17.12	AA	*Lus10005429*
Lu7_18116505	7/18116505	TRL_Index, R/S_N+	2	7.91 – 11.73	0.110	5.59	TT	-
Lu8_5060402	8/5060402	TRL_Index	2, 3	5.99 – 7.16	0.293	10.15	CC	*Lus10023890*
Lu8_6944451	8/6944451	TRL_N+, RV_N+, R/S_N+	2, 4	6.32 – 72.75	0.069	2.38	GG	-
Lu8_10951919	8/10951919	NUE-	6	4.70	0.187	7.72	CC	-
Lu8_14874163	8/14874163	PDW_Index	1	4.44	0.219	2.54	GG	-
Lu8_15171521	8/15171521	RV_N+	2	16.65	0.114	0.11	TT	-
Lu8_15393736	8/15393736	R/S_N+	2	7.51	0.065	0.46	CC	-
Lu8_17493700	8/17493700	SDW_N+	2	3.84	0.134	2.88	GG	*Lus10000482*
Lu8_17748850	8/17748850	RV_N+, TRL_N+	2, 4	4.60 – 61.66	0.333	2.71	CC	-
Lu8_19503719	8/19503719	RV_Index	2	6.70	0.191	6.60	TT	*Lus10007043*
Lu8_19661867	8/19661867	RT_N-, RT_N+	2, 4	5.35 – 5.85	0.175	4.56	TT	*Lus10014133*, *Lus10007006*
Lu8_19903484	8/19903484	TRL_Index, RT_Index	4, 6	4.76 – 4.82	0.342	3.30	AA	*Lus10024559*, *Lus10024583*, *Lus10024565*, *Lus10024571*, *Lus10024600*
Lu8_20178792	8/20178792	R/S_N+	2	40.43	0.069	1.26	AA	-
Lu8_20265310	8/20265310	RT_N-	4	5.86	0.333	5.78	AA	-
Lu8_21114435	8/21114435	RV_Index	3	4.05	0.130	5.61	CC	*Lus10033988*
Lu8_21427977	8/21427977	R/S_N-	3	5.99	0.228	4.15	AA	*Lus10034042*, *Lus10010506*
Lu8_21833452	8/21833452	R/S_N+	2	3.81	0.390	0.83	TT	*Lus10010568*, *Lus10010569*
Lu8_22089609	8/22089609	TRL_N+	2, 3	3.98 – 4.32	0.394	3.04	CC	-
Lu8_22317870	8/22317870	NUE-, RV_Index	1, 2	4.56 – 4.71	0.141	6.40	CC	*Lus10039470*, *Lus10039488*
Lu8_22556260	8/22556260	RV_N+, PDW_Index	2, 4	3.96 – 9.22	0.179	3.82	AA	*Lus10015339*, *Lus10015360*
Lu8_23500395	8/23500395	RV_N-, RT_Index	6	5.75 – 6.52	0.163	8.45	CC	*Lus10018410*
Lu9_753524	9/753524	NUE_Index	1	3.98	0.079	3.93	CC	*Lus10010187*, *Lus10010177*
Lu9_887727	9/887727	NUE_Index	2	3.85	0.085	0.07	TT	-
Lu9_1119973	9/1119973	R/S_N+, SDW_Index, PDW_Index	2, 6	4.36 – 38.70	0.374	3.45	TT	*Lus10007530*, *Lus10028970*
Lu9_1477322	9/1477322	R/S_N-	3	10.46	0.297	1.90	CC	*Lus10004338*
Lu9_1777951	9/1777951	TRL_Index	2	10.16	0.256	9.63	AA	*Lus10008972*
Lu9_3897458	9/3897458	TRL_N+	1	4.26	0.083	2.09	TT	-
Lu9_3959435	9/3959435	TRL_N+, RT_N+	1, 2	4.06 – 6.87	0.397	10.32	GG	-
Lu9_4142327	9/4142327	RV_N+	2	18.11	0.215	1.39	TT	*Lus10040365*, *Lus10040377*
Lu9_4401733	9/4401733	RV_N+	2	8.93	0.260	0.37	TT	*Lus10040297*, *Lus10040311*
Lu9_4846565	9/4846565	RV_N+	2	23.47	0.061	0.62	CC	*Lus10028267*, *Lus10028251*, *Lus10004678*
Lu9_6368499	9/6368499	NUE+	1, 3	4.51 – 4.93	0.459	16.59	AA	*Lus10031059*
Lu9_7182110	9/7182110	RV_N+	2, 6	9.20 – 60.95	0.342	3.22	GG	-

Lu9_7471774	9/7471774	R/S_N-	6	6.29	0.325	8.94	GG	-
Lu9_8326375	9/8326375	NUE_Index	2	4.28	0.069	0.02	TT	-
Lu9_8540939	9/8540939	RV_N+	2	45.44	0.293	0.25	CC	-
Lu9_9586152	9/9586152	R/S_N+	2	12.44	0.077	0.11	AA	-
Lu9_17066425	9/17066425	RV_N-	6	11.71	0.183	0.30	GG	-
Lu9_17166466	9/17166466	NUE_STI, R/S_N+	4, 6	4.24 – 5.91	0.130	5.99	GG	*Lus10022030*
Lu9_17473027	9/17473027	RV_N+, RT_N+	1, 4	3.87 – 5.98	0.115	6.94	GG	-
Lu9_17900887	9/17900887	R/S_N+	2	5.15	0.374	1.34	TT	-
Lu9_18443162	9/18443162	RV_Index	2	3.76	0.130	1.40	GG	*Lus10029683*
Lu9_19177190	9/19177190	R/S_N+, NUE_Index	2, 4	4.75 – 4.81	0.199	9.98	AA	*Lus10024873*, *Lus10024837*
Lu9_19469655	9/19469655	R/S_N-, RV_Index	2, 6	4.11 – 8.01	0.358	2.83	CC	*Lus10024901*, *Lus10024908*
Lu9_22655891	9/22655891	SDW_N-	6	4.68	0.081	1.68	TT	-
Lu10_265726	10/265726	TRL_N-	3	4.11	0.354	6.44	TT	-
Lu10_476659	10/476659	RV_N-	2	5.81	0.484	1.54	AA	*Lus10009679*
Lu10_978991	10/978991	SDW_Index, PDW_Index	4	5.07 – 7.37	0.179	9.36	AA	*Lus10031810*
Lu10_1494732	10/1494732	RV_N-, RV_N+	2, 6	4.72 – 19.10	0.386	1.08	CC	-
Lu10_1494757	10/1494757	SDW_N-, PDW_N-	2, 3, 4, 6	4.17 – 10.00	0.431	9.86	GG	*Lus10031701*, *Lus10031713*
Lu10_6500681	10/6500681	R/S_N-, R/S_N+	2, 4	3.83 – 21.45	0.203	3.73	CC	-
Lu10_10314748	10/10314748	PDW_Index, RV_N-	2, 6	4.20 – 8.00	0.203	1.27	CC	-
Lu10_11093894	10/11093894	RT_N+	4	4.09	0.138	1.79	CC	-
Lu10_11578585	10/11578585	NUE_Index	4	6.38	0.163	5.29	GG	*Lus10032750*
Lu10_13207100	10/13207100	R/S_N+	4	4.11	0.171	1.56	GG	*Lus10001130*
Lu10_13429467	10/13429467	R/S_N-	3	5.05	0.468	1.58	CC	*Lus10042679*
Lu10_14547589	10/14547589	PDW_Index	3	5.01	0.085	9.20	CC	-
Lu10_15238760	10/15238760	RV_N+	2	8.92	0.415	0.26	GG	-
Lu10_15674355	10/15674355	SDW_N-, PDW_N-, RT_N+, NUE+	1, 2, 6	5.03 – 8.12	0.314	10.51	CC	*Lus10018711*, *Lus10018756*, *Lus10018730*, *Lus10018727*, *Lus10018752*
Lu10_15998665	10/15998665	TRL_Index	1	4.41	0.488	3.82	TT	*Lus10022935*, *Lus10022924*
Lu10_16228223	10/16228223	TRL_N+	2, 4, 6	4.41 – 7.74	0.191	6.50	CC	-
Lu10_16760337	10/16760337	R/S_N+	2, 4	5.38 – 14.97	0.094	4.01	CC	*Lus10022789*
Lu10_16957730	10/16957730	SDW_N+	2	6.02	0.130	2.92	TT	*Lus10002343*, *Lus10002355*, *Lus10002353*, *Lus10002356*
Lu10_17822828	10/17822828	RV_N-	6	8.84	0.073	0.63	CC	-
Lu11_1454883	11/1454883	RT_N-	2	5.15	0.378	3.23	CC	-
Lu11_1841121	11/1841121	NUE-, NUE+, NUE_STI	2, 4	4.85 – 10.35	0.110	8.49	TT	*Lus10038783*, *Lus10038768*
Lu11_2112004	11/2112004	TRL_N-, NUE_Index	1, 4	4.34 – 5.64	0.086	3.07	GG	*Lus10038745*

Lu11_2354918	11/2354918	SDW_Index, PDW_Index, R/S_N+	1, 2, 4, 6	9.22 – 32.98	0.118	13.35	GG	-
Lu11_3027697	11/3027697	PDW_N-	6	7.56	0.447	3.11	CC	-
Lu11_3158486	11/3158486	PDW_N+	2, 6	4.01 – 4.41	0.382	1.89	GG	-
Lu11_3337135	11/3337135	SDW_N-, PDW_N-, RT_N-, RT_N+, RV_N+	1, 2, 4, 6	4.25 – 58.68	0.120	5.86	CC	*Lus10001245*
Lu11_3725133	11/3725133	RV_Index	2, 6	3.86 – 5.21	0.110	3.46	GG	-
Lu11_4178266	11/4178266	RV_N-	6	4.48	0.065	2.82	TT	*Lus10026400*
Lu11_4239182	11/4239182	TRL_N_, RV_N-, RV_N+	1, 2, 4, 6	5.63 – 16.47	0.228	10.80	AA	-
Lu11_4454464	11/4454464	RV_N+	2	20.92	0.496	0.70	GG	-
Lu11_5325891	11/5325891	TRL_Index, RV_Index	1, 2, 4, 6	3.83 – 7.15	0.272	7.22	AA	-
Lu11_5349141	11/5349141	RT_N-	4	7.82	0.077	10.74	TT	-
Lu11_5595131	11/5595131	RV_N-, RV_N+	2, 6	16.23 – 30.83	0.350	0.85	GG	*Lus10038251*
Lu11_6444164	11/6444164	PDW_Index	4	5.70	0.183	7.33	TT	-
Lu11_14898826	11/14898826	NUE-	6	4.64	0.317	11.36	TT	*Lus10008564*
Lu11_15604119	11/15604119	R/S_N+	2	42.15	0.138	0.34	TT	-
Lu11_15873610	11/15873610	TRL_Index	1	4.32	0.252	4.74	GG	-
Lu11_16222926	11/16222926	R/S_N+	6	6.52	0.098	4.86	CC	*Lus10009900*
Lu11_16959462	11/16959462	NUE_Index	6	6.59	0.081	5.10	TT	*Lus10039313*, *Lus10039324*, *Lus10039317*
Lu11_17231523	11/17231523	RT_Index	2, 4, 6	4.94 – 8.72	0.386	7.24	TT	*Lus10039278*
Lu11_17749943	11/17749943	SDW_Index	4	4.41	0.325	2.80	GG	*Lus10031655*, *Lus10031652*
Lu12_308207	12/308207	RV_N+, NUE-, NUE+	1, 2, 4, 6	4.24 – 26.81	0.266	14.05	GG	*Lus10019979*, *Lus10019970*, *Lus10019975*, *Lus10019985*
Lu12_418795	12/418795	RV_Index	1	7.60	0.265	9.32	GG	*Lus10019979*, *Lus10020002*, *Lus10019985*, *Lus10020006*
Lu12_721567	12/721567	RV_N+	2	22.00	0.293	0.46	CC	*Lus10006777*
Lu12_1020497	12/1020497	TRL_Index	4, 6	4.36 – 5.43	0.260	4.40	AA	*Lus10036176*
Lu12_1371982	12/1371982	RV_N-	6	10.41	0.264	1.09	GG	-
Lu12_1386203	12/1386203	RT_N+, R/S_N-	2, 4	4.82 – 5.10	0.268	5.26	TT	*Lus10036251*
Lu12_1606069	12/1606069	PDW_N+	2	5.96	0.333	4.63	TT	-
Lu12_1841489	12/1841489	RV_N+	2	15.40	0.244	2.93	CC	-
Lu12_2350248	12/2350248	RT_N-, RT_Index, NUE_Index	1, 2, 3, 4, 6	3.70 – 9.98	0.496	7.53	GG	*Lus10023238*, *Lus10023230*
Lu12_4187854	12/4187854	RT_Index	3	4.49	0.439	13.44	CC	*Lus10016554*
Lu12_4334157	12/4334157	PDW_N+	1	6.09	0.339	12.69	CC	*Lus10016554*, *Lus10016545*

Lu12_4546209	12/4546209	R/S_N+	2	25.64	0.350	0.29	GG	-
Lu12_5158397	12/5158397	R/S_N+	2	25.14	0.398	0.13	GG	*Lus10018301*
Lu12_6465853	12/6465853	RV_N+	2	70.89	0.431	1.78	CC	*Lus10016819*, *Lus10016833*
Lu12_14028030	12/14028030	RT_Index	2, 4	3.85 – 6.26	0.211	6.24	AA	-
Lu12_14495719	12/14495719	R/S_N-	6	14.52	0.191	3.66	AA	-
Lu12_15997644	12/15997644	R/S_N-, R/S_N+	2, 6	5.57 – 11.44	0.110	10.48	GG	-
Lu12_18307372	12/18307372	RV_N+, R/S_N-	2, 4	4.50 – 17.03	0.439	2.33	TT	*Lus10034156*
Lu12_18519589	12/18519589	RV_Index	2, 4, 6	6.22 – 10.15	0.098	10.02	GG	-
Lu12_18713836	12/18713836	SDW_Index, PDW_Index, NUE+, R/S_N-	2, 3, 6	4.33 – 8.20	0.382	3.69	AA	*Lus10003058*
Lu12_19289913	12/19289913	SDW_Index	2	4.83	0.146	2.54	AA	*Lus10033664*
Lu12_20223199	12/20223199	R/S_N+	2, 4, 6	4.12 – 26.81	0.130	2.91	AA	-
Lu13_28309	13/28309	TRL_Index	1	4.72	0.190	6.55	CC	-
Lu13_340653	13/340653	RV_N+	2	33.67	0.281	0.49	AA	-
Lu13_492674	13/492674	RV_N+	2	10.63	0.236	0.33	CC	-
Lu13_720051	13/720051	TRL_N+, RV_N+	2, 6	3.87 – 43.82	0.220	5.77	TT	*Lus10008052*
Lu13_820912	13/820912	PDW_N-	1	4.68	0.285	4.49	TT	-
Lu13_874776	13/874776	RV_N+	2	31.67	0.232	0.63	GG	-
Lu13_1763895	13/1763895	RT_N+	4	4.53	0.089	3.18	AA	*Lus10001338*
Lu13_2852677	13/2852677	PDW_N+	6	6.07	0.199	2.02	GG	-
Lu13_3455092	13/3455092	R/S_N-	2, 6	5.65 – 6.03	0.358	2.68	CC	*Lus10026061*
Lu13_4010608	13/4010608	RT_N-	2	5.10	0.224	5.17	AA	*Lus10002739*, *Lus10002741*
Lu13_5496902	13/5496902	NUE-, NUE+, NUE_STI	1, 2, 4	3.73 – 8.89	0.270	11.07	CC	*Lus10011530*
Lu13_5626614	13/5626614	R/S_N+	2	37.20	0.146	0.16	AA	*Lus10011576*
Lu13_5925834	13/5925834	PDW_N-	4	3.84	0.061	6.33	TT	*Lus10004297*, *Lus10004302*, *Lus10004289*
Lu13_7087309	13/7087309	RT_Index	1	5.89	0.438	23.07	CC	-
Lu13_8092006	13/8092006	PDW_Index	1	5.29	0.347	12.18	GG	-
Lu13_10246937	13/10246937	TRL_Index	2	5.57	0.256	4.27	AA	-
Lu13_10595042	13/10595042	RV_Index	2	5.79	0.374	5.41	AA	-
Lu13_10711791	13/10711791	TRL_Index, RV_Index	4, 6	4.15 – 5.20	0.358	5.63	GG	-
Lu13_11127657	13/11127657	PDW_N-, RV_N+	1, 2	4.11 – 13.27	0.169	2.66	GG	*Lus10032866*
Lu13_11656001	13/11656001	R/S_N-	6	6.71	0.122	2.73	GG	-
Lu13_13257585	13/13257585	NUE+	6	5.07	0.175	2.28	AA	-
Lu13_14182845	13/14182845	RV_N+	2	26.35	0.167	0.59	CC	-
Lu13_17243516	13/17243516	NUE+	4	9.28	0.114	10.82	TT	*Lus10025586*

Lu13_17528763	13/17528763	PDW_Index	2	4.38	0.122	4.14	TT	-
Lu13_18363934	13/18363934	RT_Index	2, 4	5.22 – 13.33	0.301	5.30	GG	*Lus10030902*, *Lus10030878*
Lu13_19124582	13/19124582	RV_N+, R/S_N+	2, 4	4.40 – 99.03	0.163	2.39	GG	*Lus10030488*, *Lus10030483*
Lu13_19396336	13/19396336	NUE_STI	2, 6	6.47 – 7.02	0.146	13.13	AA	-
Lu13_19514686	13/19514686	SDW_N-, PDW_N-, RV_N-, NUE-, SDW_N+, PDW_N+, TRL_N+, RV_N+, RT_N+	1, 2, 3, 4, 6	3.75 – 82.77	0.231	12.85	TT	-
Lu13_19525774	13/19525774	TRL_N-, RV_N-, RT_N-	1, 2, 3, 4, 6	5.12 – 13.32	0.122	8.45	AA	-
Lu13_19862281	13/19862281	TRL_Index	1	9.15	0.066	10.73	GG	*Lus10011352*, *Lus10011346*
Lu13_20238784	13/20238784	R/S_N+	2	11.29	0.130	1.80	TT	*Lus10002581*
Lu14_361008	14/361008	R/S_N+	2	38.69	0.094	0.20	TT	*Lus10009468*
Lu14_893430	14/893430	NUE+	4	4.49	0.085	4.99	CC	-
Lu14_1151456	14/1151456	R/S_N+, NUE_Index	2, 3	4.28 – 46.92	0.342	0.58	CC	*Lus10028627*
Lu14_1541988	14/1541988	SDW_N+, PDW_N+	6	5.72 – 7.51	0.187	2.27	GG	*Lus10028695*, *Lus10028679*
Lu14_1999537	14/1999537	NUE-, NUE_STI	2	5.04 – 5.56	0.309	4.92	AA	-
Lu14_2032660	14/2032660	NUE+	2	11.36	0.228	15.46	AA	-
Lu14_2349386	14/2349386	SDW_Index, PDW_Index	4, 6	3.87 – 6.33	0.130	3.87	GG	-
Lu14_2844536	14/2844536	RT_N+	6	5.62	0.285	2.28	TT	-
Lu14_3036652	14/3036652	NUE-, NUE_STI	2, 3, 6	3.77 – 5.76	0.342	6.62	CC	*Lus10004876*, *Lus10004872*
Lu14_3630064	14/3630064	SDW_Index, PDW_Index	2, 6	3.74 – 4.10	0.122	4.33	CC	-
Lu14_3838919	14/3838919	SDW_Index, PDW_Index, R/S_N-	2, 4, 6	4.03 – 8.90	0.150	14.79	CC	*Lus10021422*, *Lus10021428*
Lu14_4290395	14/4290395	NUE_Index	4	4.67	0.146	1.18	GG	*Lus10021475*
Lu14_4771319	14/4771319	RV_N-, SDW_N+	2, 6	3.88 – 4.11	0.077	3.93	CC	*Lus10012517*, *Lus10012519*
Lu14_4932491	14/4932491	SDW_N+, PDW_N+, NUE+	1, 2, 6	5.04 – 7.35	0.484	4.11	TT	-
Lu14_5215316	14/5215316	RV_N+	2	33.70	0.260	0.65	CC	*Lus10014158*
Lu14_5729675	14/5729675	SDW_Index, PDW_Index	3	4.39 – 5.60	0.484	9.55	CC	-
Lu14_6225285	14/6225285	SDW_N+, PDW_N-, PDW_N+	2, 6	5.57 – 10.82	0.175	4.28	CC	-
Lu14_9866805	14/9866805	RV_N-	6	5.65	0.281	2.40	GG	*Lus10004760*, *Lus10008192*
Lu14_10356043	14/10356043	RT_N-	2, 3	4.70 – 5.36	0.301	5.50	CC	*Lus10003748*, *Lus10003740*

Lu14_12738805	14/12738805	SDW_Index, PDW_Index	2, 6	4.14 – 5.90	0.211	6.47	TT	-
Lu14_14781992	14/14781992	SDW_N-	2	4.10	0.187	2.73	GG	-
Lu14_15168783	14/15168783	SDW_N+	2	6.61	0.480	5.28	GG	*Lus10035730*
Lu14_15396478	14/15396478	TRL_N+, RT_N-, RT_N+	1, 2, 6	3.94 – 8.29	0.140	9.75	CC	*Lus10035654*, *Lus10035664*, *Lus10035673*
Lu14_15526556	14/15526556	RV_N+, NUE_STI, TRL_N-, RV_N-	1, 2, 4, 6	3.98 – 7.71	0.134	4.79	GG	*Lus10035654*, *Lus10035628*, *Lus10003231*
Lu14_15927781	14/15927781	RV_N+	2	14.14	0.163	0.32	TT	*Lus10042154*, *Lus10042127*, *Lus10042128*
Lu14_17132252	14/17132252	R/S_N+	2	4.94 – 7.81	0.244	1.71	AA	*Lus10005574*, *Lus10005578*
Lu14_17573678	14/17573678	R/S_N+	2	13.42	0.118	0.43	TT	-
Lu14_17971007	14/17971007	NUE_Index	2	6.12	0.098	1.19	GG	*Lus10039041*
Lu15_60022	15/60022	SDW_Index	2	6.91	0.203	4.77	AA	-
Lu15_504983	15/504983	RV_N+, TRL_N+, RV_N-	1, 2, 6	5.80 – 41.95	0.112	12.73	AA	*Lus10002596*, *Lus10002599*
Lu15_868802	15/868802	R/S_N-	6	4.89	0.142	2.94	AA	*Lus10011239*
Lu15_5135728	15/5135728	TRL_Index	1	6.66	0.231	13.46	CC	-
Lu15_7036901	15/7036901	TRL_N+, RV_N+	2, 4, 6	4.57 – 76.29	0.089	3.58	TT	*Lus10001481*, *Lus10001479*
Lu15_7286304	15/7286304	RV_N-	6	5.90	0.439	1.16	GG	*Lus10005987*, *Lus10005997*
Lu15_8398899	15/8398899	R/S_N+	2, 6	6.91 – 34.58	0.301	3.24	GG	*Lus10040916*
Lu15_8939174	15/8939174	R/S_N-, TRL_Index	1, 3, 4	3.95 – 9.60	0.146	6.21	CC	*Lus10040999*, *Lus10041030*, *Lus10041037*
Lu15_9126429	15/9126429	SDW_N+, TRL_Index	1, 4	4.14 – 6.37	0.343	16.55	TT	*Lus10041044*, *Lus10041072*
Lu15_9428955	15/9428955	RV_N+	2	53.30	0.211	3.10	AA	-
Lu15_9529977	15/9529977	RV_N+	2	40.65	0.236	0.61	AA	*Lus10041153*
Lu15_9732109	15/9732109	NUE+	6	5.06	0.285	1.95	TT	-
Lu15_9760082	15/9760082	R/S_N+	2	19.69	0.069	0.57	GG	-
Lu15_10090727	15/10090727	NUE+, SDW_N-	2	4.05 – 6.11	0.220	4.64	AA	*Lus10041309*, *Lus10041285*
Lu15_10375834	15/10375834	NUE-, NUE_STI	2	4.27 – 5.49	0.171	7.38	CC	-
Lu15_10407306	15/10407306	NUE_STI	1, 4	3.93 – 5.96	0.160	10.33	CC	-
Lu15_10539015	15/10539015	R/S_N+	2	4.34	0.122	2.90	GG	*Lus10041402*
Lu15_11179267	15/11179267	TRL_Index	2, 4	6.05 – 6.30	0.276	4.52	TT	-
Lu15_13172607	15/13172607	RV_N+	2	6.12	0.317	0.71	GG	*Lus10010286*, *Lus10010284*
Lu15_13590145	15/13590145	RT_Index	2	5.82	0.110	6.25	AA	*Lus10009967*
Lu15_13664294	15/13664294	SDW_Index, PDW_Index	2, 6	4.50 – 7.89	0.077	6.75	CC	-
Lu15_13811574	15/13811574	NUE-	3	4.32	0.317	6.75	AA	*Lus10037978*
Lu15_14075063	15/14075063	R/S_N+	2	12.07	0.065	0.45	TT	*Lus10037925*
Lu15_14211627	15/14211627	TRL_N-, RV_N-	2, 6	4.54 – 4.99	0.106	5.64	CC	*Lus10037892*, *Lus10037894*

Lu15_14451884	15/14451884	SDW_Index, PDW_Index	2, 3, 4	4.08 – 7.46	0.402	8.87	CC	-
Lu15_14701381	15/14701381	RV_N+	2	19.96	0.195	1.97	TT	-

*1, 2, 3, 4, 5, and 6 represent mrMLM, FASTmrMLM, FASTmrEMMA, ISIS EM-BLASSO, pKWmEB, and pLARmEB, respectively. Bold text indicates pleiotropic QTL. MAF = Minor allele frequency > 0.05

Table S4. Summary of read numbers and mapped reads from the RNA-Seq analysis

Sample ID	Raw reads	HQ reads	%HQ	Number of mapped reads	% mapped (from hq reads)
HN-N0-R1	26186612	25634568	98.07	24026638	91.75
HN-N0-R2	31440357	30754943	97.98	29123044	92.63
HN-N0-R3	31591373	30902313	97.98	28951668	91.64
HN-N0-R4	23958564	23444602	97.97	22092449	92.21
HN-N1-R1	30771046	30125083	98.03	28168431	91.54
HN-N1-R2	33547421	32808879	97.96	30490536	90.89
HN-N1-R3	33866998	33158610	98.06	30784145	90.90
HN-N1-R4	21871133	21448333	98.23	19980893	91.36
LN-N0-R1	22384872	21953587	98.22	20731804	92.62
LN-N0-R2	25810407	25196537	97.79	23513530	91.10
LN-N0-R3	21399235	20955647	98.11	19551670	91.37
LN-N0-R4	22296996	21870222	98.24	20458320	91.75
LN-N1-R1	21309014	20826784	97.88	19387170	90.98
LN-N1-R2	21864092	21470869	98.36	19844818	90.76
LN-N1-R3	22994955	22583208	98.35	21266395	92.48
LN-N1-R4	20003344	19599262	98.11	18052452	90.25

HN: High NUE accession O_IRL_C_CN98192, LN: Low NUE accession O_IND_C_CN98982. N0: Depleted nitrogen treatment, N1: Optimum nitrogen treatment. HQ: High quality.
